# The *Sinocyclocheilus* cavefish genome provides insights into cave adaptation

**DOI:** 10.1186/s12915-015-0223-4

**Published:** 2016-01-04

**Authors:** Junxing Yang, Xiaoli Chen, Jie Bai, Dongming Fang, Ying Qiu, Wansheng Jiang, Hui Yuan, Chao Bian, Jiang Lu, Shiyang He, Xiaofu Pan, Yaolei Zhang, Xiaoai Wang, Xinxin You, Yongsi Wang, Ying Sun, Danqing Mao, Yong Liu, Guangyi Fan, He Zhang, Xiaoyong Chen, Xinhui Zhang, Lanping Zheng, Jintu Wang, Le Cheng, Jieming Chen, Zhiqiang Ruan, Jia Li, Hui Yu, Chao Peng, Xingyu Ma, Junmin Xu, You He, Zhengfeng Xu, Pao Xu, Jian Wang, Huanming Yang, Jun Wang, Tony Whitten, Xun Xu, Qiong Shi

**Affiliations:** State Key Laboratory of Genetic Resources and Evolution, Kunming Institute of Zoology, Chinese Academy of Sciences, Kunming, 650223 China; BGI-Shenzhen, Shenzhen, 518083 China; Shenzhen Key Lab of Marine Genomics, State Key Laboratory of Agricultural Genomics, Shenzhen, 518083 China; Fauna & Flora International, Cambridge, CB1 2JD UK; China National Genebank, Shenzhen, 518083 China; Agricultural Genomes Institute at Shenzhen, Chinese Academy of Agricultural Sciences, Shenzhen, 518120 China; College of Life Sciences, University of Chinese Academy of Sciences, Beijing, 100049 China; School of Life Science and Technology, University of Electronic Science and Technology of China, Chengdu, 610054 China; BGI-Yunnan, Kunming, 650106 China; Shenzhen BGI Fisheries Sci & Tech Co. Ltd., Shenzhen, 518083 China; Zhenjiang BGI Fisheries Science & Technology Industrial Co. Ltd., Zhenjiang, 212000 China; Shanghai Synchrotron Radiation Facility, Shanghai Institute of Applied Physics, Chinese Academy of Sciences, Shanghai, 201204 China; State Key Laboratory of Reproductive Medicine, Department of Prenatal Diagnosis, Nanjing Maternity and Child Health Care Hospital, Nanjing Medical University, Nanjing, 210029 China; Freshwater Fisheries Research Center, Chinese Academy of Fishery Sciences, Wuxi, 214081 China; James D. Watson Institute of Genome Science, Hangzhou, 310008 China; Department of Biology, Ole Maaløes Vej 5, University of Copenhagen, DK-2200 Copenhagen, Denmark

**Keywords:** Cavefish, Genome, Adaptation, Evolution, Qinghai-Tibetan Plateau, *Sinocyclocheilus*

## Abstract

**Background:**

An emerging cavefish model, the cyprinid genus *Sinocyclocheilus*, is endemic to the massive southwestern karst area adjacent to the Qinghai-Tibetan Plateau of China. In order to understand whether orogeny influenced the evolution of these species, and how genomes change under isolation, especially in subterranean habitats, we performed whole-genome sequencing and comparative analyses of three species in this genus, *S. grahami*, *S. rhinocerous* and *S. anshuiensis*. These species are surface-dwelling, semi-cave-dwelling and cave-restricted, respectively.

**Results:**

The assembled genome sizes of *S. grahami*, *S. rhinocerous* and *S. anshuiensis* are 1.75 Gb, 1.73 Gb and 1.68 Gb, respectively. Divergence time and population history analyses of these species reveal that their speciation and population dynamics are correlated with the different stages of uplifting of the Qinghai-Tibetan Plateau. We carried out comparative analyses of these genomes and found that many genetic changes, such as gene loss (e.g. opsin genes), pseudogenes (e.g. crystallin genes), mutations (e.g. melanogenesis-related genes), deletions (e.g. scale-related genes) and down-regulation (e.g. circadian rhythm pathway genes), are possibly associated with the regressive features (such as eye degeneration, albinism, rudimentary scales and lack of circadian rhythms), and that some gene expansion (e.g. taste-related transcription factor gene) may point to the constructive features (such as enhanced taste buds) which evolved in these cave fishes.

**Conclusion:**

As the first report on cavefish genomes among distinct species in *Sinocyclocheilus*, our work provides not only insights into genetic mechanisms of cave adaptation, but also represents a fundamental resource for a better understanding of cavefish biology.

**Electronic supplementary material:**

The online version of this article (doi:10.1186/s12915-015-0223-4) contains supplementary material, which is available to authorized users.

## Background

As one of the most successful vertebrate colonizers in subterranean habitats, cavefishes attract interest because of their unusual regressive features, such as the rudimentary eyes and scales, and loss of pigmentation. As possible compensation, some constructive traits have evolved, such as elongated appendages and non-visual sensory systems [1, 2]. Nevertheless, biologists have long puzzled over these troglomorphic characters [3], and the study of their relationship to their environment has been largely ignored [4]. Although recent work on a traditional model, *Astyanax mexicanus*, revealed some important advances [3], especially in aspects of eye loss [5] and identifying candidate genes underlying quantitative trait loci (QTL) [6], there is no whole genomic data available to unravel the evolution and general adaptation to dark subterranean life among other groups of distinct but closely-related cavefishes.

**Fig. 1:**
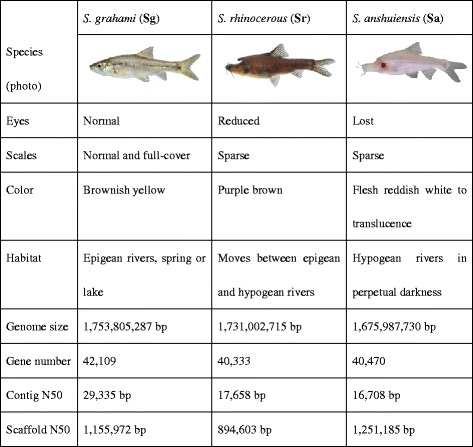
Comparison of biological traits, habitat and basic genomic information for the three sequenced *Sinocyclocheilus* species. They are representative of surface-dwelling (Sg), semi-cave-dwelling (Sr) and cave-restricted (Sa) species, respectively. Please note the regressive characters in the adult Sa, such as loss of eyes, little scale covering and translucent skin

*Sinocyclocheilus* (Cypriniformes: Cyprinidae) is endemic to China’s massive southwestern karst area, the northern part of which abuts the eastern part of the Qinghai-Tibetan Plateau. This genus serves as an emerging cavefish model for its high species diversity and the phenotypic variation which has evolved in karst river systems. In a previous *Science* letter in 2013 [7], we reported a global hotspot of biodiversity and the threats to it through a survey of cave species in Southwestern China, with an illustration of a *Sinocyclocheilus* cavefish, *S. rhinocerous*, which was collected from Yunnan province. The present study deals with whole genome and transcriptome sequencing of three *Sinocyclocheilus* species (Fig. 1): the surface-dwelling *S. grahami* (Sg); the semi-cave-dwelling *S. rhinocerous* (Sr); and the cave-restricted *S. anshuiensis* (Sa). These species were carefully chosen as representatives of three key nodes on the path to obligate cave life, although almost all *Sinocyclocheilus* species show some cave-related traits and habits [8]. We investigate whether whole genomic data would provide some clues about genetic adaptations to subterranean habitats, and whether the strict co-occurrence of the karstic landscape and *Sinocyclocheilus* species might serve to reveal aspects of the biogeographic history of the uplift of the Qinghai-Tibetan Plateau.

## Results and discussion

### Genome assembly, assessment and annotation

High-quality genomic DNA was extracted from muscle tissues of the three *Sinocyclocheilus* species, which were collected from Yunnan (Sg and Sr) and Guangxi (Sa) provinces in China (Additional file 1: Figure S1). A series of sequencing libraries (250 bp to 20 kb) were constructed and applied in a whole-genome shotgun sequencing strategy, and a total of 313.3, 174.0 and 188.2 Gb of raw data were obtained for the Sg, Sr and Sa fishes, respectively (Additional file 2: Table S1). The assembled genome sizes are approximately 1.7 Gb (1.75 Gb for Sg, 1.73 Gb for Sr and 1.68 Gb for Sa), and the calculated contig N50 and scaffold N50 values are 17–29 Kb and 0.9–1.3 Mb, respectively (Fig. 1; Additional file 2: Table S3). The quality of the three genome assemblies was evaluated (Additional file 2: Tables S5 and S9, Additional file 3: Figures S6 and S7), and our assessment confirmed that all were high in quality and could be used for further comparative analyses. In addition, we employed a standard annotation pipeline to predict gene sets, resulting in approximately 40,000 genes in the three fish genomes (42,109 for Sg, 40,333 for Sr and 40,470 for Sa; see more details in Additional file 2: Table S15 and Additional file 4: Figure S14), which were double that of most diploid species (Additional file 4: Figure S13). The further Hox gene distribution analyses in three *Sinocyclocheilus* species (Additional file 4: Figure S11) and genome alignment between zebrafish and Sg (Additional file 4: Figure S10) provided more evidence to support the tetraploid nature of *Sinocyclocheilus*.

### Phylogenetic relationships and divergence time

**Fig. 2:**
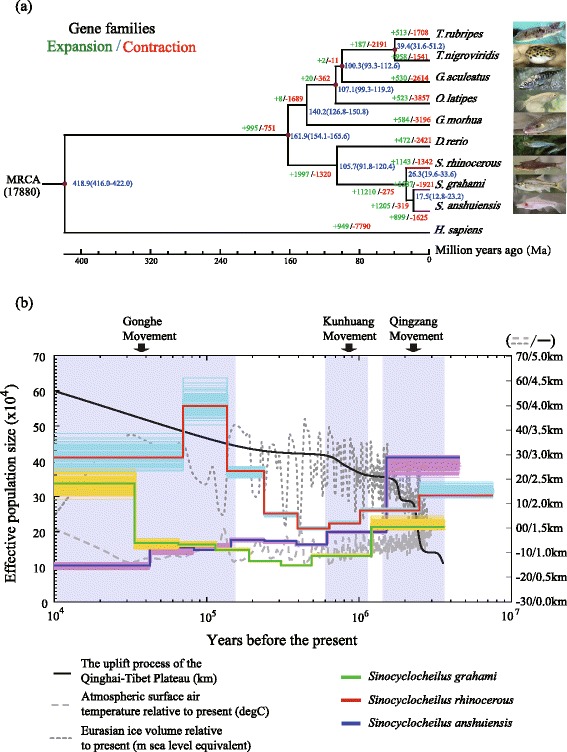
Phylogenomic analysis and demographic histories of the three *Sinocyclocheilus* species. **a** Phylogenomic relationships inferred from 3,181 orthologous genes of the three *Sinocyclocheilus* and six other teleost species (*Homo sapiens* was the outgroup), with the branch lengths scaled to estimated divergence times (numbers in blue show median and range values). The numbers besides the branch indicated expanded (green) and contracted (red) gene families since the split from a most recent common ancestor (MRCA). **b** Demographic histories were reconstructed using the pairwise sequentially Markovian coalescent (PSMC) model. The uplift process of the Qinghai-Tibet Plateau since 3.6 Ma was obtained from a published paper [11] and other significant environmental events, such as the atmospheric surface air temperature and Eurasian ice volume for the past 3 Ma, were taken from the National Centers for Environmental Information (NCEI; http://www.ncdc.noaa.gov/). The time range of three rounds of intense uplift (Qingzang, Kunhuang and Gonghe Movement) is highlighted in lilac. All three species have similar patterns of decrease under the Qingzang and Kunhuang Movement, but a subsequent divergent trend under the Gonghe Movement

The collision between India and Asia in the early Cenozoic may have been the largest-ever orogenic event in the Earth’s history [9]. It not only triggered the extraordinary uplift of the Qinghai-Tibet Plateau [10], but also profoundly influenced the Asian climate through the genesis of the Asian monsoon [11], the development of large-scale drainage patterns [12], and especially, the speciation and biodiversity of the organisms living on and below the Plateau (e.g. [9, 13]). Because *Sinocyclocheilus*, the most species-rich cyprinid genus, is endemic to the massive southwestern karst area (the northern part of which abuts the eastern part of the Qinghai-Tibetan Plateau), it has been proposed that the development of this diversity was correlated with the cyclic uplift and planation of the Qinghai-Tibet Plateau [14, 15]. Both the phylogenetic analyses of single-copy orthologous genes and of the mitochondrial DNA datasets (Additional file 5: Figures S15 and S16) supported this correlation, recovering the same relationships for the three *Sinocyclocheilus* species studied here (Fig. 2a). The generated chronogram revealed that Sr emerged first from the other two species at 26.3 Ma, and Sg divided from Sa at 17.5 Ma. These divergence times are similar to those obtained for other speciation events of congeners inferred from a more comprehensive study of Cyprininae phylogeny [16]. These splitting events occurred during the initial and final phases, respectively, of the second tectonic uplift of the Qinghai-Tibet Plateau (25–17 Ma), a time when evidence shows that the limestone region they lived in had been experiencing large-scale karst development [17]. The frequent capture and isolation of the subterranean river systems along with the karst development were regarded as the key reasons for speciation in this genus [15], although the complete picture will remain unknown until more species have been examined. The ancient separation revealed here might be one of the reasons to explain the high species diversity within a relatively narrow distribution area (about 270,000 km^2^ [15]) (Additional file 1: Figure S1).

### Population history

A reconstruction of the population demography of the three *Sinocyclocheilus* species has revealed a similar start but a subsequent divergent trend from 10^4^ to 10^7^ years ago (Fig. 2b). It seems that the population demography has a greater correlation with the uplifting of the Qinghai-Tibet Plateau than other significant environmental events, such as the Eurasian ice volume or the atmospheric surface air temperature. In a relatively short period of time (from 3.0 to 0.5 Ma), all three species underwent two rounds of population decline, which occurred following two intense uplift phases in the third tectonic uplift of the Qinghai-Tibet Plateau [17, 18]. These two phases of movements at Qingzang (3.6–1.7 Ma) and Kunhuang (1.1–0.6 Ma) uplifted the Plateau from an average of <1,000 m up to 4,000 m, followed by the intensification of the Asian monsoon and increased precipitation [18], in addition to the large-scale development of glaciers [17]. The patterns of coincident population declines in the three species are believed to be a response of a common background following the intense uplift of the Plateau that may have been unfavorable to them (although it is still not clear which kind of environmental changes contributed the most). However, two events of unusual population expansion were also detected after these events. One was recognized in Sr during the interglacial period (0.5–0.15 Ma), a period during which Sg and Sa kept their low populations, which hints at the presence of extensive subterranean water systems in the Sr-native Luoping Basin at this time. Another was observed for Sg from 0.023 Ma when its main distribution area, the paleo-lake Dianchi, was coincidentally documented from geological sediments to have been some three times larger than at present [19]. Range expansion along new river channels might accompany Plateau uplift, thereby providing some fish the opportunity to increase their population sizes as reported in the typical Plateau schizothoracine fish *Schizopygopsis pylzovi* [20] and *Gymnocypris chilianensis* [21].

### Variations of eye structures and related genetic and expression responses

**Fig. 3:**
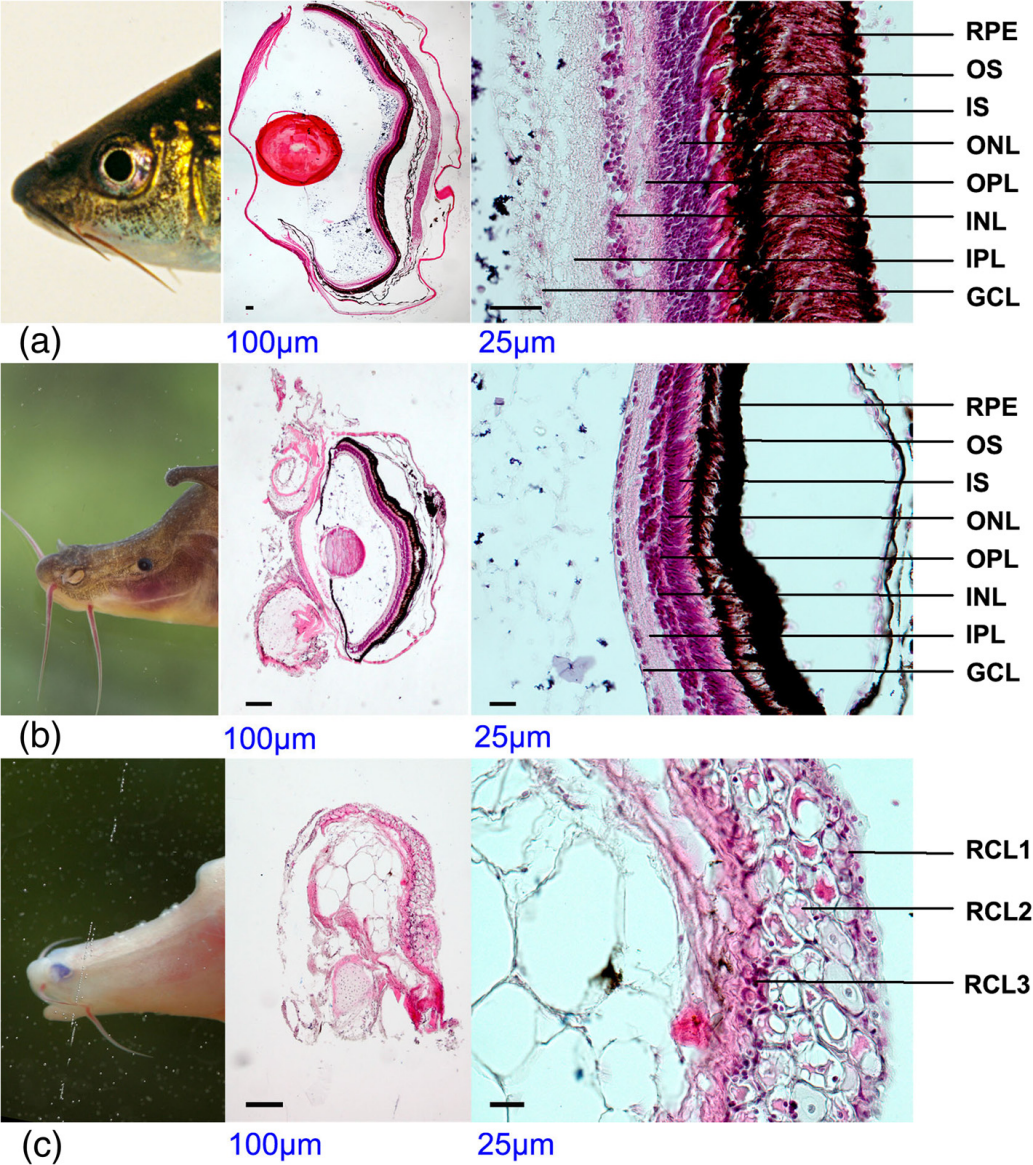
Comparison of retinal structures among the three *Sinocyclocheilus* species. Phenotypes and H&E stained sections of eyes from top to bottom are those in **a** Sg, **b** Sr and **c** Sa, respectively. GCL, ganglion cell layer; INL, inner nuclear layer; IPL, inner plexiform layer; IS, inner segment; ONL, outer nuclear layer; OPL, outer plexiform layer; OS, outer segment; RCL, relict cell layer; RPE, retinal pigmented epithelium

Cavefishes often display regressive features, such as the degeneration of the eyes, which often develop normally during embryogenesis but subsequently arrest, degenerate or sink beneath the skin [22]. In the three adult *Sinocyclocheilus* fishes in this study, Sg has normal-sized eyes with a lens and retinal structures; Sr has small eyes with a reduced lens and retinal cell density; and Sa has lost its external eyeballs and lens, and the retina has degenerated to become merely disorganized and indistinguishable cellular layers (Fig. 3). The recently published *A. mexicanus* genome identified many specific candidate genes under eye-related QTL, and revealed expression differences in several development stages [6]. In our study, we analyzed the genetic changes of these existing crystallin and opsin genes and also presented copy number variations among cave-dwelling and surface-dwelling fishes. Our comparative genomic data suggested that several opsin genes, including *Lws2* (long wavelength-sensitive), *Rh2-1* and *Rh2-2* (middle wavelength-sensitive), have been lost in *Sinocyclocheilus*, and that *Rh2-4* has been lost in Sa (Additional file 2: Table S26). In dim conditions, long wavelength light is rapidly attenuated [23], and it seems rational that the long and middle wavelength-sensitive genes were lost specifically in Sa. The results from transcriptomic analyses of eyes also unsurprisingly showed significantly lower expression levels of most visual opsin genes in Sa when compared with Sg and Sr (Additional file 2: Table S27). Development and maintenance of photoreceptors requires a series of transcriptional factors. As expected, nine important transcriptional factors, including *Crx*, *Nrl*, *Otx2*, *Otx5*, *Nr2e3*, *Ggca1A*, *Gnat1*, *Gnat2* and *Rorb*, were significantly down-regulated in Sa (Additional file 2: Table S27 and Additional file 6: Figure S25). These results are consistent with those found for *Astyanax* cavefishes [24] and for other *Sinocyclocheilus* species [25, 26], which supports the hypothesis that down-regulation of rhodopsin might play a critical role in eye degradation of cavefishes [25, 26].

By screening crystallin genes in the three *Sinocyclocheilus* genomes, we found that copy numbers of most genes were lower than those of the diploid zebrafish (Additional file 2: Table S26 and Additional file 6: Figure S28). Transcriptomic data further demonstrated that the expression levels of most crystallin genes were maintained at high levels in Sg, but were not expressed in Sa (Additional file 2: Table S27). We also observed that several crystallin genes in Sa (such as *Cryball1*, *Crygm2d12* and *Crygm7*) have evolved into pseudogenes due to existing premature stop codons (Additional file 2: Table S25E). Previous studies in *Astyanax* found that the expression of crystallin genes were down-regulated in the development of cavefish lens [27], suggesting that the lens plays a critical role in promoting cell survival in the development of eyes [28]. In particular, *β*- and *γ-*crystallins play a pivotal role in retinal tissue remodeling and repair, and also strongly enhance axon regeneration of retinal ganglion cells [29]. Hence, the lack, or down-regulation, of crystallin gene expression in Sa supports its phenotype of a visual system in which both the lens and retina have degenerated. Interestingly, we also found that Sa has two copies of *Hsp90α1.1* and *Hsp90a1.2* (heat shock protein 90*α*) genes, while both Sg and Sr have only one. Meanwhile, the expression levels of *Hsp90α* in Sa eyes were much higher than those in Sg and Sr (Additional file 2: Table S27). These observations provide further evidence to support a novel role of *Hsp90α* in lens apoptosis and eye degeneration of cavefishes [30].

### Different mechanisms of albinism compared with *Astyanax*

In order to check the mechanisms of loss of pigmentation in Sa skin, we compared melanogenesis-related genes in the three *Sinocyclocheilus* genomes. In *Astyanax*, the albinism of some cave populations is caused by the deletions of *Oca2* gene exon regions, which disturbs the upstream steps of the melanin synthesis pathway [31]. Although similar deletions of *Oca2* genes were not found in either of the two copies in Sa (Additional file 6: Figure S21), nor were any identical mutations specifically present in Sa and *Astyanax* cave populations, some other new mutations were identified (Additional file 6: Figure S21), and the transcriptome analysis performed on the skin of Sg, Sr and Sa indicated that the expression of *Oca2* gene in Sa was the lowest (Additional file 2: Table S20). In the melanin synthesis pathway, several enzymes work downstream, such as Tyr (tyrosinase), a key rate-limiting enzyme, and Tyrp1 (tyrosinase-related protein 1) [32]. More interestingly, we found that Tyr has an amino acid mutation (*G420R*) in Sa (Additional file 6: Figure S18), which was identical to that identified in Caucasian human patients (*G419R*, http://www.ifpcs.org/albinism/oca1mut.html) [33, 34]. Meanwhile, the expression levels of Sa genes in the melanogenesis pathway in the skin transcriptomes were the lowest, especially *Tyr* and *Tyrp1*, with significantly lower expression levels in Sa compared with Sg (Additional file 2: Table S20). It seems that similar phenotypes in *Sinocyclocheilus* cavefish might evolve by different mechanisms from *Astyanax*. Furthermore, we found that the Mpv17 protein in Sa had a deletion in the signal region compared with zebrafish, Sg and Sr (Additional file 6: Figure S22), and the expression level of *Mpv17* in Sa is the lowest (Additional file 2: Table S20). A previous study shows that the deletion in *Mpv17* can cause the *tra* mutant phenotype in zebrafish, and cause a loss (or strong reduction) of iridophores throughout larval and adult stages [35]. This study also pointed out that differentiated iridophores were required for the accumulation and maintenance of melanophores during pigment pattern formation [35], and a parallel study showed that the interaction between iridophores and other chromatophores is critical in the stripe formation of zebrafish [36]. Thus we infer that the deletion of *Mpv17* might cause the loss of iridophores, which affected the formation of melanophores, and consequently played a role in the albinism of Sa.

### Gene mutation and loss in scale degeneration

A previous study has indicated that mutations in the ectodysplasin-A receptor (*Edar*) encoding locus can lead to complete scale loss in fish such as medaka [37]. For this reason, two copies of *Edar* gene (named as *Edar1* and *Edar2*, respectively) in the three *Sinocyclocheilus* genomes were identified and checked. Interestingly, one of the proteins, Edar1, has deletions in the signal peptide and partial extracellular regions in all three *Sinocyclocheilus* species, which may lead to functional changes or loss in this copy (Additional file 6: Figure S20). For the other protein copy, Edar2, only Sa has the signal peptide region and partial extracellular regions totally deleted when compared with Sg and Sr (Additional file 6: Figure S20). This deletion in Sa may lead to a functional disorder in guiding the Edar protein transfer across the membrane, thus generating fewer scales at the skin surface of Sa (Additional file 7: Figure S29). Coincidentally, two important genes, *Lamb3* and *Col7a*, were also lost from the Sa genome, which may cause a defect in the anchoring between the epidermis and dermis, resulting in friction and skin fragility [38, 39] in the scale covering.

### Possible hearing loss in Sa

**Fig. 4:**
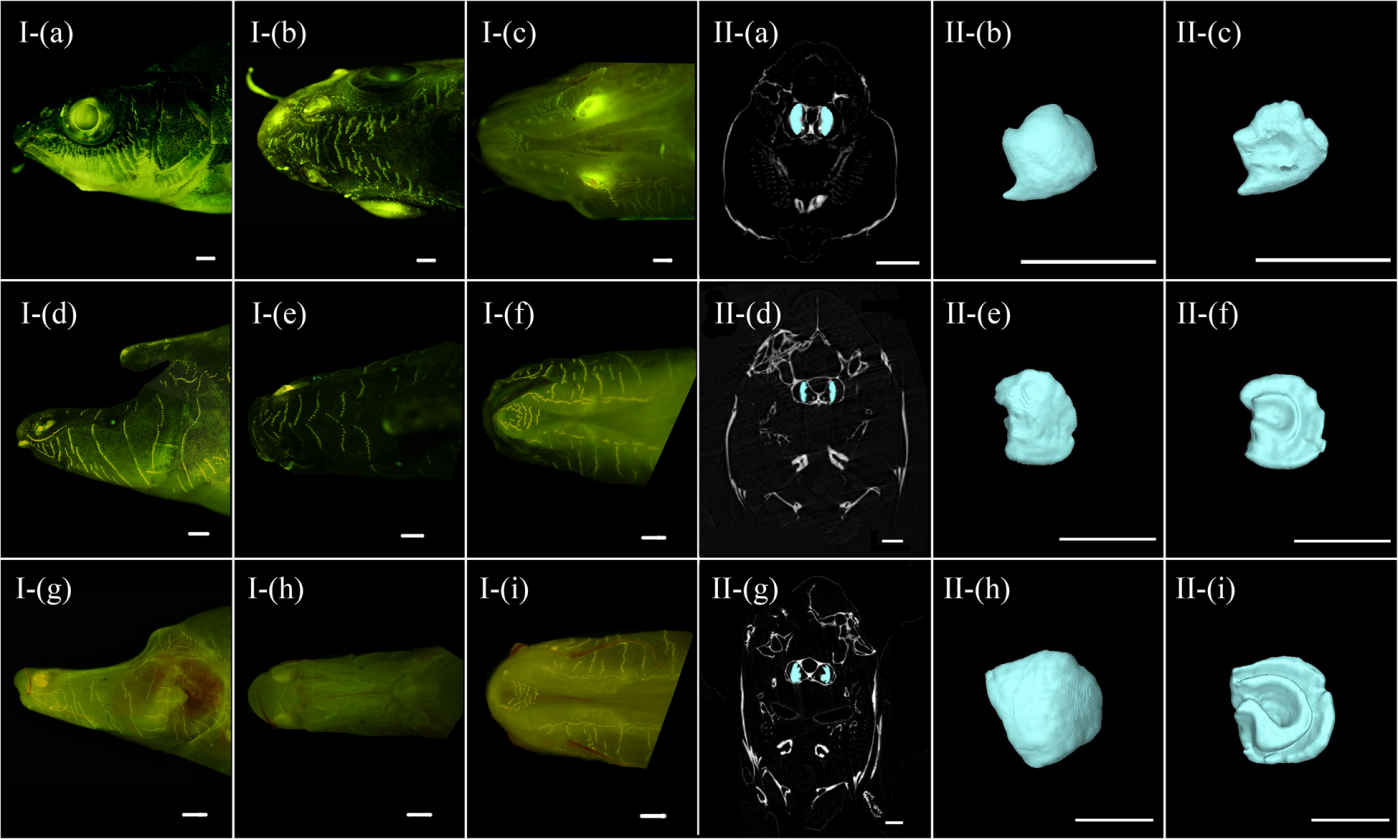
**I** The distributions of superficial neuromasts on the head and **II** morphology of saccular otolith in the inner ear among the three *Sinocyclocheilus* species. The superficial neuromasts after DASPEI staining from the plates I-(a–c), I-(d–f) and I-(g–i) represent Sg, Sr and Sa, respectively. The photos from left to right show the lateral view, dorsal view and ventral view. These figures show that the numbers of neuromasts in the adult fishes decline in the following order: Sg > Sr > Sa. The morphology of the saccular otoliths was reconstructed based on synchrotron X-ray microtomography. The plates II-(a–c), II-(d–f) and II-(g–i) represent Sg, Sr and Sa, respectively. The photos from left to right show the location of saccular otoliths in the inner ear, the dorsal view and ventral view of its morphology. The ventral of saccular otolith in Sa is seriously aberrant, with a deep and expanded central pit, encircled by another lateral sulcus. The degree of corrosion increase is in the following order: Sg < Sr < Sa. Scale bar: 1 mm

The hearing of cavefishes is interesting but less studied than the other functions discussed above. We found the deletion of *Mpv17* gene, which we mentioned in the section on albinism, may also have some influence in hearing. A previous study reported that *Mpv17*-deficient mice suffered from degeneration of cochlea and loss of sensorineural hearing at 2 months old [40]. Another gene, *Ush2a*, has also changed in Sa, especially two amino acid sites, *R334S* [41, 42] and *V382A* [43] (referred to the human sites), which may affect splicing and the termination codon (Additional file 6: Figure S19). It has been proved that the encoded protein of *Ush* is found in the basement membrane and may be related to the development of the inner ear [44, 45]. These mutations in Sa may cause its sensorineural deafness. There are no similar changes in Sg and Sr (Additional file 6: Figure S22). Reconstructions of the saccular otolith morphology using synchrotron X-ray microtomography among these three species show that the ventral surface of this otolith in Sa is seriously aberrant, and the degree of corrosion has distinctly increased in the following order: Sg < Sr < Sa (Fig. 4). Both the anatomy of the swim bladder (Additional file 7: Figure S30) and the numbers of neuromasts and scales of the trunk lateral line system (Additional file 7: Figure S31) also indicate these hearing-related organs in Sa have different degrees of weakness. Our data together indicated that the cave-restricted species Sa might have reduced hearing, which could be similar with that demonstrated in amblyopsid cavefishes [46]. Furthermore, a comparison of the distribution of neuromasts on the head (Fig. 4) also suggested that the response to vibration of these three species was Sg > Sr > Sa, which is different from the general pattern in *Astyanax* [47].

### Different immune responses to specific habitats

The immune activities of Sa may be lower than its epigean counterparts because it probably lives in a less diverse microbial environment. The fact that Sa is more susceptible to disease in captivity might support this inference (unpublished data). We found relatively fewer copies of immune genes in Sa when compared with those in Sg and Sr (Additional file 2: Table S22). However, one important innate immune group, the *Tlr* (toll-like receptor) gene family, showed some degree of expansion in Sa (Additional file 2: Table S23), through the duplication of *Tlr8* and *Tlr18* in the Sa genome. This suggests that these three species may have evolved differential immune activities for innate immunity and adaptive immunity according to their different habitats. Interestingly, the semi-cave-dwelling Sr had substantially more copy numbers of immune genes than Sg and Sa (Additional file 2: Table S22), which may be a result of an adaptation to heterogeneous elements between epigean and hypogean habitats, as found in the amphibious mudskippers [48].

### Lack of diurnal rhythms

Previous research reported that some cavefishes lack diurnal rhythms when living in perpetual darkness [49]. For example, cave populations of *A. mexicanus* have a phenotype of reduced sleep in comparison to their surface relatives [50]. We found that both the two copies of *Skp1* in Sa had deletions in the N-terminal end of its protein (Additional file 6: Figure S24). Since Skp1 is one of the components of the Skp1-Cul1-Fbxl3 (SCF) protein complex, and the SCF complex is most relevant in the mammalian clock mechanism [51], the deletions in Skp1 might lead to some dysfunction in SCF, which suggests weaker light rhythms in Sa. Meanwhile, the transcriptomic analysis of the eye demonstrated that expression levels of the rhythm pathway genes decrease in the order Sg > Sr > Sa (Additional file 2: Table S28 and Additional file 6: Figure S26).

### Low fecundity in Sa

Although low fecundity is often assumed to be normal in cave species [4], there is little empirical evidence, and the different fecundity levels between surface and cave forms sometimes seem to be habitat plastic (e.g. [52]). We know of no study on the fecundity of the cave-dwelling *Sinocyclocheilus* species. Our analysis of the absolute fecundity (number of mature eggs) of Sg and Sa (2,402.9 ± 881.9 in Sg [53] vs. 143 ± 116 in Sa (count from four specimens with mature eggs in this study)) indicated that the fecundity in cave *Sinocyclocheilus* species is much less than surface congeners. Interestingly, one related gene, *Creb3l4*, was found to have been lost in the Sa genome. It has been reported that *Creb3l4* can regulate the expression of genes required for germ cell survival, although it is insufficient to disrupt the normal fertility in mice [54].

### Enhancement of taste

Taste buds are enhanced in some cavefishes, such as *Astyanax* [55]. We applied the taste-related gene sequences of zebrafish to BLAST to the three *Sinocyclocheilus* genomes, and unexpectedly found that one taste receptor gene, the *Tast1r2-1*, was significantly expanded (almost fourfold) and one important transcription factor, the *Prox1* gene, had threefold copies in these three species compared with zebrafish (Additional file 8: Table S29). However, if these expansions were correlated to the overall enhancement of taste in the three *Sinocyclocheilus* species (benthic and cave-preferred) we still need further testing. Some taste receptor genes, such as *Tas1r1* and *Tas2r200-2*, were specifically duplicated in the Sa genome (vs. Sg and Sr) (Additional file 8: Table S29), which suggests a further improvement in the sense of taste in the cave-restricted Sa. A preliminary study of the distribution of taste buds within the jaws of these three species also indicated that their numbers increase in a sequence from Sg < Sr < Sa (Additional file 7: Figure S32).

## Conclusion

**Fig. 5:**
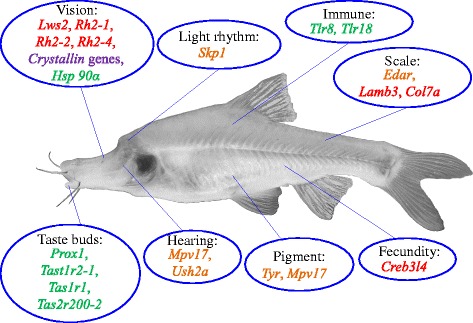
Summary of the most important genetic changes in the cave-restricted Sa. The main results are outlined as follows: *Lws2*, *Rh2-1*, *Rh2-2* and *Rh2-4* are lost in Sa. Several crystallin genes, including *Crygmx* in the Sr and *Cryball1*, *Crygm2d2*, *Crygm7* and *Crygmx* in Sa, have evolved into pseudogenes. Sa has two *Hsp90α* genes while Sg and Sr have only one; meanwhile, the expression of *Hsp90α* in Sa eyes is higher than that in Sg and Sr. *Mpv17* has a deletion in the signal region in the Sa genome. *Ush2a* has two amino acid changes, i.e. *R334S* and *V382A. Tyr* has a nucleotide mutation (*G420R*) in one copy of the Sa genome. Two copies of *Edar* gene in Sa represent deletions, and *Lamb3* and *Col7a* were lost. Two copies of *Skp1* protein in Sa have deletions in the N-terminal end. *Prox1* and *Tast1r2-1* are under expansions in the three *Sinocyclocheilus* species genomes, and *Tas1r1* and *Tas2r200-2* are specifically duplicated in the Sa genome. Red, gene loss; green, gene expansion; purple, pseudogene; orange, mutation or deletion

This paper focused on comparative genomic and transcriptomic studies of three *Sinocyclocheilus* species, representative of surface-dwelling (Sg), semi-cave-dwelling (Sr) and cave-restricted (Sa) species. We found that speciation and population dynamics of these fishes are closely related to the uplifting stages of the Qinghai-Tibetan Plateau. Comparative genome analysis revealed many genetic changes, such as gene loss, pseudogenes, mutations and down-regulation, which were associated with regressive features (eye degeneration, albinism, rudimentary scales and low fecundity), and some gene expansions suggest that constructive features (such as the sense of taste) have evolved in caves (a few supplementary notes can be seen in Additional file 9: Note S6). The most important genetic changes in the cave-restricted Sa are summarized in Fig. 5. Among them, some changes are similar to those in other cavefishes (e.g. *A. mexicanus*) from very different parts of the world (such as the down-regulation of some rhodopsin-related genes), but some are not (such as without vs. with the exon deletion of *Oca2* gene in Sa vs. some *A. mexicanus* cave populations). Although the incidence of gene losses and expansions in cave-restricted Sa corresponds to the distinct phenotype variations, the changes seem to be fixed in its genome rather than in other mechanisms. The genetic changes found at the genome level in this study, although needing further functional confirmation, still give some good clues to understanding the mysteries of cave adaptation.

In the history of cave biology research, people have often been confused by the plethora of views and terms, such as disuse, preadaptation, opportunism, compensation, regressive evolution, etc. Adaptations to cave living are complicated because there is no morphological ‘archetype’ for cave animals, and the characters show a highly diverse mix [4]. There is still a long way to go before this work can be applied to general evolutionary theory. As the first report on cavefish genomes among distinct species in *Sinocyclocheilus*, our work provides not only insights into genetic mechanisms of cave adaptation, but is also a fundamental resource for better understanding of cavefish biology.

## Methods

### Genome sequencing and assembly

Three female adult *Sinocyclocheilus* fishes, collected from Yunnan province (Sg and Sr) and Guangxi province (Sa) of China, were used for sequencing. The research protocol and treatment of experimental fishes have been reviewed and approved by the internal review board of the Kunming Institute of Zoology, Chinese Academy of Sciences (approval ID: SYDW-2014020). High-quality genomic DNA was extracted from muscle tissues using Puregene Tissue Core Kit A (Qiagen, MD, USA) for construction of libraries with different inserted sizes (250 bp to 20 kb) (Additional file 2: Table S1). In total, 25 paired-end libraries (11 for Sg, 7 for Sr and 7 for Sa, respectively) were generated with the Illumina standard operating procedure. HiSeq 2000 shotgun sequencing and assembling by SOAPdenovo assembler were performed as previously reported [56]. Artificial and low-quality reads were filtered first and then sequencing errors with 17-mer frequency lower than four were collected using a method described in a previous study [57]. Next, 88.05-, 48.31- and 60.54-fold coverage of *Sinocyclocheilus* genomes were used for assembly (Additional file 2: Table S2). In addition, we estimated genome sizes of the three *Sinocyclocheilus* fishes using the 17-mer depth frequency distribution method: G (Genome size) = K-mer_num/Peak_depth. The estimated genome sizes are 1.79, 1.89 and 1.81 Gb, respectively (Additional file 2: Table S4). Subsequently, the filtered reads were assembled into contigs and scaffolds with SOAPdenovo [58] and gaps were fulfilled with GapCloser [58]. Finally, a primary assessment was performed on the genome assemblies (Additional file 9: Note S1).

### Genome annotation

We performed homology-based predictions by running RepeatMasker (version 3.3.0) [59] against the RepBase [60], and identified repeat sequences at DNA and protein levels using TE library (version 16.10) and RepeatProteinMask. We searched the *de novo* prediction build repeat library using RepeatModeler (version 1.0.5) and generated TE results with classification information for each repeat family by running RepeatMasker on the genome sequences subsequently. Tandem repeats were also searched using Tandem Repeats Finder (version 4.04) [61]. The protein coding genes were obtained using a combination of the *de novo* method, homology-based gene prediction and RNA-Seq data (Additional file 9: Note S2). All predicted gene evidence was integrated by GLEAN [62] to get non-redundant data [63].

### Transcriptome analysis

RNA was isolated for sequencing from four tissues (eye, skin, liver and ovary) of Sg, Sr and Sa, respectively. We applied an in-house C++ program to filter raw reads and then obtain high-quality reads (Additional file 9: Note S3). All of the clean RNA-Seq reads were mapped onto the corresponding reference genomes (Sg, Sr and Sa) using TopHat (version 2.0.4) [64]. According to the mapped results, transcripts were constructed using Cufflinks (version 2.0.0) [65]. The *de novo* transcriptomes of the four tissues were assembled by Trinity with filtered reads from each tissue separately into contigs and scaffolds. Trinity contains Inchworm, Chrysalis and Butterfly, which were employed sequentially to process large volumes of RNA-Seq reads.

### Evolutionary analysis

(**1**) Gene family cluster: we defined gene families using TreeFam (http://www.treefam.org) among the three *Sinocyclocheilus* fishes (Sg, Sr and Sa) and seven other vertebrates, including fugu (*Takifugu rubripes*), green spotted puffer (*Tetraodon nigroviridis*), three-spined stickleback (*Gasterosteus aculeatus*), Atlantic cod (*Gadus morhua*), medaka (*Oryzias latipes*), zebrafish (*Danio rerio*) and human (*Homo sapiens*). A total of 17,883 gene families and 210 single-copy gene families were identified. The numbers of orthologous genes across the ten species were counted (Additional file 2: Table S19 and Additional file 4: Figure S13) and plotted in a Venn diagram (Additional file 4: Figure S14). (**2**) Phylogenetic analysis: phylogenetic relationships were established using 3,181 single-copy orthologous genes shared among nine teleost fish genomes (Additional file 5: Figure S15) using maximum likelihood (ML) method in PhyML [66, 67] and Bayesian inference method in MrBayes [68]. An additional dataset of six mitochondrial gene sequences were also used to reconstruct the phylogenetic trees (Additional file 5: Figure S16). (**3**) Divergence time estimation: the divergence times were estimated using the mcmctree [69] in PAML [70] and recalculated using the multidivtime [71, 72] program, and all were calibrated by five fossil records [73] (Additional file 5: Figure S17 and Additional file 9: Note S4). (**4**) Demographic history: the distribution of time to TMRCA (the most recent common ancestor) between two alleles in an individual can be related to the history of population size fluctuation. The population size histories of Sg, Sr and Sa were inferred using the pairwise sequentially Markovian coalescent (PSMC) model [74] on heterozygous sites with the generation time (g = 1 year) (according to artificial breeding of Sg) and the mutation rate (m = 3.51 × 10^−9^ per year per nucleotide) [75]. Reconstructed population history was plotted for Sg, Sr and Sa separately using gnuplot (version 4.4) [76]. In addition, we obtained the atmospheric surface air temperature (°C) and Eurasian ice volume (m sea level equivalent) data for the past 3 million years from the NCEI (http://www.ncdc.noaa.gov/). (**5**) Gene family contraction and expansion: we performed *Sinocyclocheilus* lineage-specific expansion and contraction analysis using the CAFE program [77]. Based on random birth and death model [78], a global parameter λ was estimated using maximum likelihood. Comparing each branch and their ancestor branch, we calculated a conditional *P* value and marked families with a *P* value of less than 0.05 as a significant change [79], which means it underwent contraction or expansion during evolution. These families were then subjected to GO/KEGG/IPR enrichment analyses along each *Sinocyclocheilus* lineage, respectively (Additional file 2: Table S25). More details are shown in Additional file 8: Table S29. (**6**) Genes with accelerated evolutionary rate: positive Darwinian selection at the DNA sequence level has been tested by estimating the ratio (ω) of nonsynonymous nucleotide substitutions (dN) to synonymous nucleotide substitutions (dS) between ortholog genes [80]. A branch-site model was used to search for the positive selection genes (PSG) [81]. After obtaining the *Sinocyclocheilus* PSG list (Additional file 10: Table S30), we converted it to the corresponding human orthologs as the input against a background of human genes [80] using the DAVID Functional Annotation [82] tool. (**7**) Evolution of Hox clusters: to define Hox genes in the three *Sinocyclocheilus* genomes, the Hox genes of zebrafish were downloaded from the ensemble as cross-references. The Hox gene numbers (Additional file 2: Table S18) and the order along the scaffolds (Additional file 4: Figure S11) in *Sinocyclocheilus* indicated that these fishes are indeed tetraploids when compared to diploid zebrafish. (**8**) Loss of Sa-specific gene families: in order to identify Sa-specific gene family loss, we extracted gene families that have no member in Sa while more than zero in the other nine species. The lost gene family list is included in Additional file 2: Table S24.

### Morphological comparison

(**1**) Paraffin sections of the eyes of three *Sinocyclocheilus* species: histological studies on eyes were performed from paraffin sections and hematoxylin and eosin (H&E) staining. (**2**) Immunocytochemistry of taste buds: this analysis focused on distributions of taste buds on upper and lower jaws, using a primary antibody (rabbit against calretinin, labeling entire receptor cells within taste buds) according to a standard protocol (Additional file 9: Note S5). (**3**) Anatomy of gas bladder, absolute fecundity and other measurements: all samples for anatomy and measurements were from specimens immersed in 90 % ethanol. (**4**) Synchrotron X-ray microtomography of the saccular otolith: this experiment was performed at BL13W1 beamline at the Shanghai Synchrotron Radiation Facility (Shanghai, China); the slices were reconstructed using FBP algorithm, and 3D renderings were created and manipulated in VGStudio 2.1 software.

### Data availability

The Whole Genome Shotgun projects have been deposited at GenBank under accession numbers LCYQ00000000 (Sg), LAVF00000000 (Sr) and LAVE00000000 (Sa), which are the same versions described in this paper. The RNA-Seq data from four tissues (eye, skin, liver and ovary) have been deposited at GenBank under accession numbers SRS1179797–SRS1179800 (Sg), SRS1179996–SRS1179999 (Sr) and SRS1180000–SRS1180003 (Sa).

## Additional files

Additional file 1: Figure S1. The collection sites of three *Sinocyclocheilus* species in this study. (PDF 415 kb) Additional file 2: Table S1–S28. The supplementary data and statistics, including the information of genome sequencing and assembly (Table S1–S9), genome annotation (Table S10–S17), evolutionary analyses (Table S18–S24), and cave adaption analysis (Table S25–S28). (XLSX 134 kb) Additional file 3: Figures S2–S7. The characteristics of genomes, including the 17-mer frequency distribution (Figure S2), GC content distributions (Figure S3), correlation of GC content and sequencing depth (Figure S4), sequencing depth (Figure S5), comparison of the assembled Sg genome with three fosmid sequences (Figure S6), and conserved sequences (Figure S7). (PDF 468 kb) Additional file 4: Figure S8–S14. The characteristics of repeats and genes, including the distributions of TEs (Figure S8), mRNA, CDS, exon and intron lengths (Figure S9), double-conserved synteny between Dr and Sg genomes (Figure S10), the evolution of Hox genes (Figure S11) and the similarity analysis (Figure S12), protein orthology comparison (Figure S13), and shared orthologous groups among Dr and three *Sinocyclocheilus* genomes (Figure S14). (PDF 666 kb) Additional file 5: Figure S15–S17. The phylogenetic trees and divergence time analyses, including the phylogenetic trees based on CDS, protein, 1st, 2nd, 1st and 2nd, 3rd, 4d codon position of codon sequences of 3,181 genes (Figure S15), mitochondrial CDS sequences (Figure S16), and divergence times calibrated by five fossil records (Figure S17). (PDF 170 kb) Additional file 6: Figure S18–S28. Analyses to the specific genes, including *Tyr* (Figure S18), *Ush2a* (Figure S19), *Edar* (Figure S20), *Oca2* (Figure S21), *Mpv17* (Figure S22), *Rom1b* (Figure S23), *Skp1* (Figure S24), and differential expression of eye-related genes, transcriptional factors (Figure S25) and circadian rhythm pathway genes (Figure S26), and similarity cluster of the *Tlr* gene family (Figure S27) and crystallins (Figure S28). (PDF 1295 kb) Additional file 7: Figure S29–S32. Morphological analyses of the three *Sinocyclocheilus* species, including scales (Figure S29), swim bladder (Figure S30), lateral line system (Figure S31) and taste buds (Figure S32). (PDF 434 kb) Additional file 8: Table S29. The results of gene family contraction and expansion. (XLSX 75 kb) Additional file 9:
**Notes S1–S6.** The supplementary notes to the article, including the organism background, genome sequencing and assembly (Note S1), genome annotation (Note S2), transcriptome analysis (Note S3), evolutionary analyses (Note S4), morphological comparison analysis (Note S5), and cave adaption analysis in the aspects of vision, pigmentation, scale development, hearing, immune response, circadian rhythm and sense of taste (Note S6). (PDF 668 kb) Additional file 10: Table S30. The *Sinocyclocheilus* positive selection gene (PSG) list. (XLSX 153 kb)
