# Supplementary material for: The Sinocyclocheilus cavefish genome provides insights into cave adaptation
Source: BMC Biol. 2016 Jan 4;14:1. doi: 10.1186/s12915-015-0223-4 (PMC4698820; doi:10.1186/s12915-015-0223-4)
Supplement: Additional file 1: Figure S1. — The collection sites of three Sinocyclocheilus species in this study. (PDF 415 kb) [file 12915_2015_223_MOESM1_ESM.pdf]

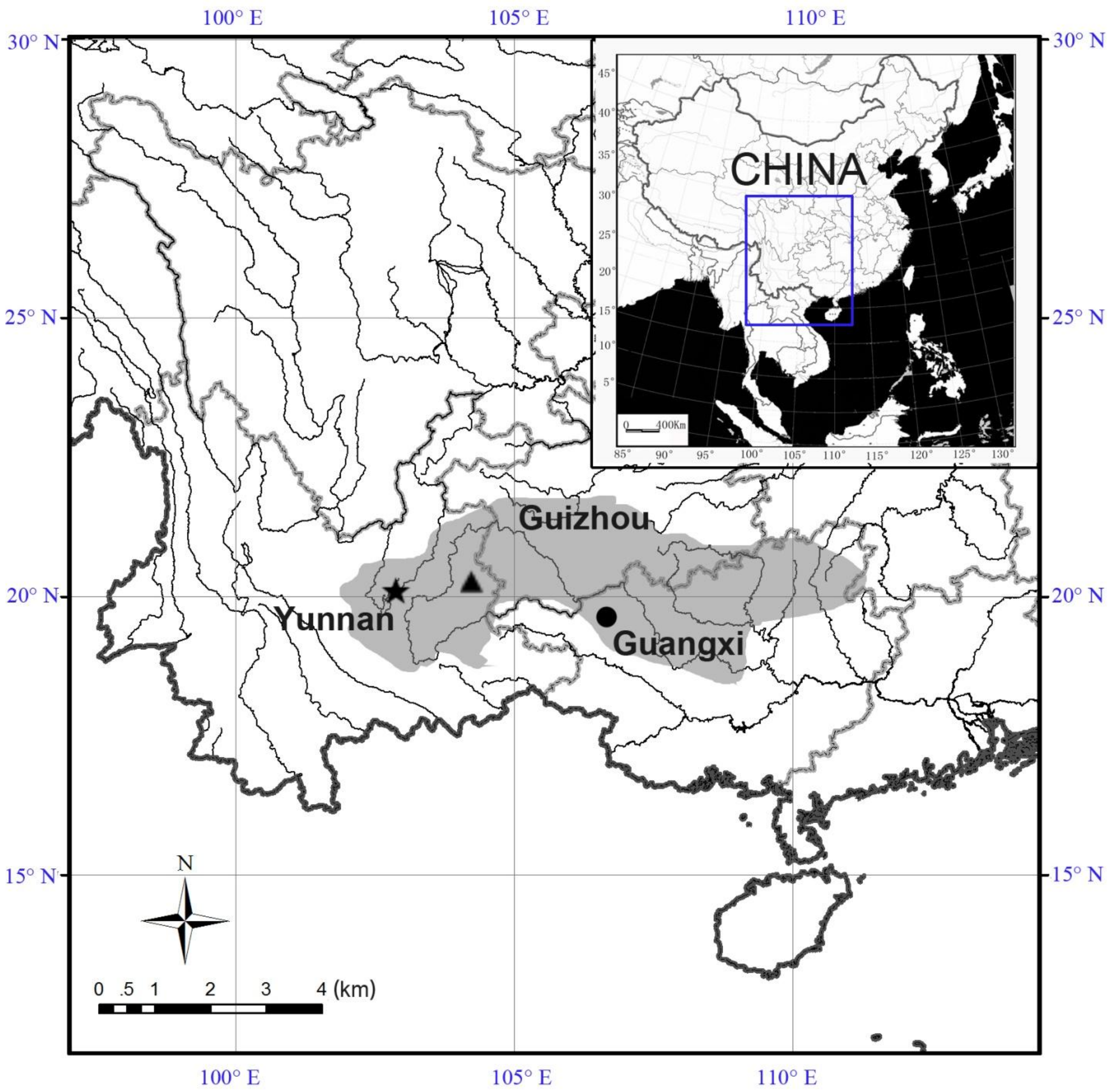

Figure S1. The collection sites of three *Sinocyclocheilus* species in this study. ★: Sg (*S. grahami*), ▲: Sr (*S. rhinocerous*), ●: Sa (*S. anshuiensis*). The shaded area (about 270,000 km^2) shows the entire distribution of the genus *Sinocyclocheilus* according to [1].
