# Supplementary material for: The Sinocyclocheilus cavefish genome provides insights into cave adaptation
Source: BMC Biol. 2016 Jan 4;14:1. doi: 10.1186/s12915-015-0223-4 (PMC4698820; doi:10.1186/s12915-015-0223-4)
Supplement: Additional file 3: Figures S2–S7. — The characteristics of genomes, including the 17-mer frequency distribution (Figure S2), GC content distributions (Figure S3), correlation of GC content and sequencing depth (Figure S4), sequencing depth (Figure S5), comparison of the assembled Sg genome with three fosmid sequences (Figure S6), and conserved sequences (Figure S7). (PDF 468 kb) [file 12915_2015_223_MOESM3_ESM.pdf]

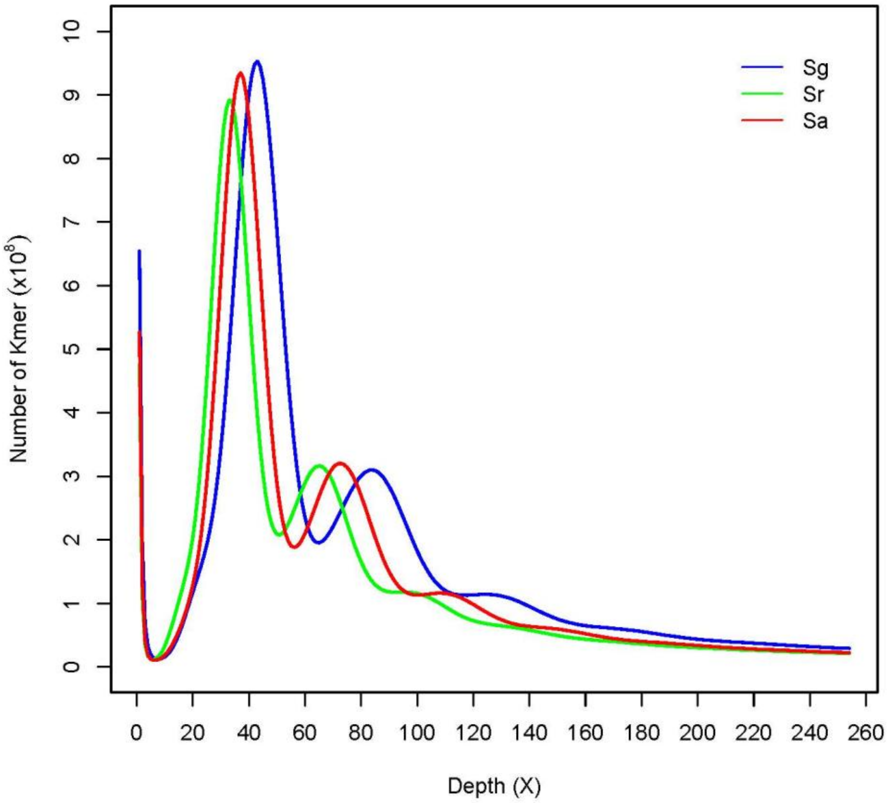

Figure S2. 17-mer frequency distribution in the three *Sinocyclocheilus* genomes. The lines show the peak depth in the sequenced fishes: Sg, 43; Sr, 37; Sa, 42. Existence of the second peaks indicates that all of the three genomes have a fairly high proportion of repetitive sequences.

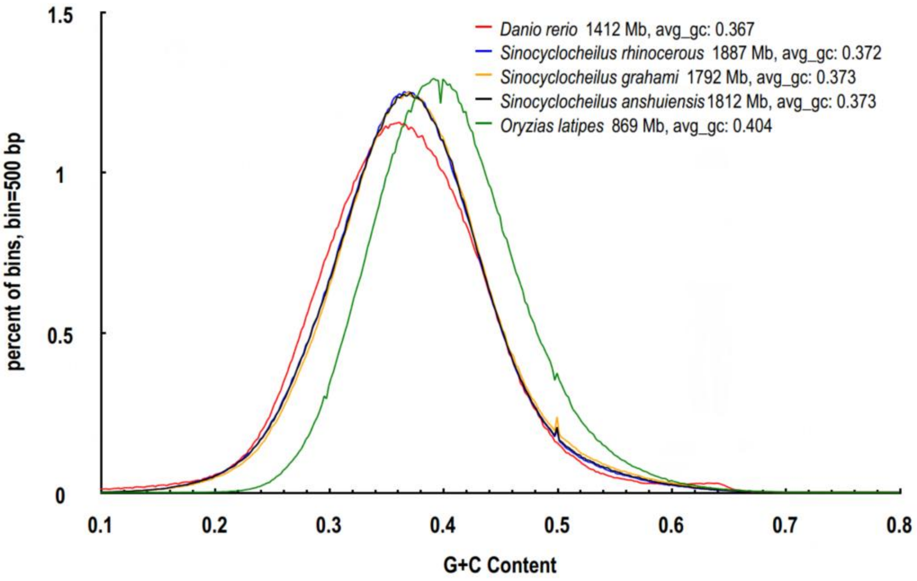

Figure S3. GC content distributions in the three *Sinocyclocheilus* genomes. The x-axis is GC content and the y-axis is the proportion of the bin numbers divided by the total windows. We used 500-bp bins (with a 250-bp overlap) sliding along the genomes. Our data demonstrate that the three genomes have similar GC content distributions.

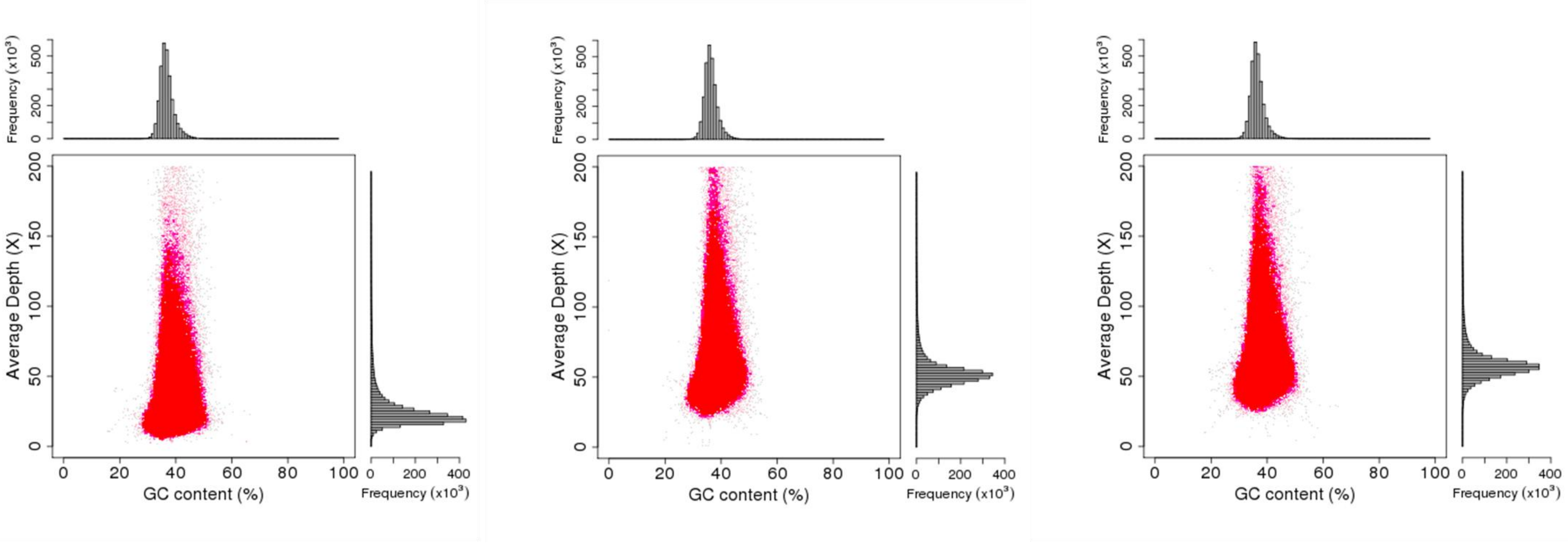

Figure S4. Correlation of GC content and sequencing depth. The x-axis represents GC content and the y-axis represents the average depth. The scatter diagram in the middle shows GC content and sequencing depth. The histogram on the top shows the GC content percentage and the histogram on the right shows the sequencing depth percentage. We used 10-kb non-overlapping sliding windows and calculated the GC content and average depth among the windows. (a): Sg, (b): Sr, (c): Sa.

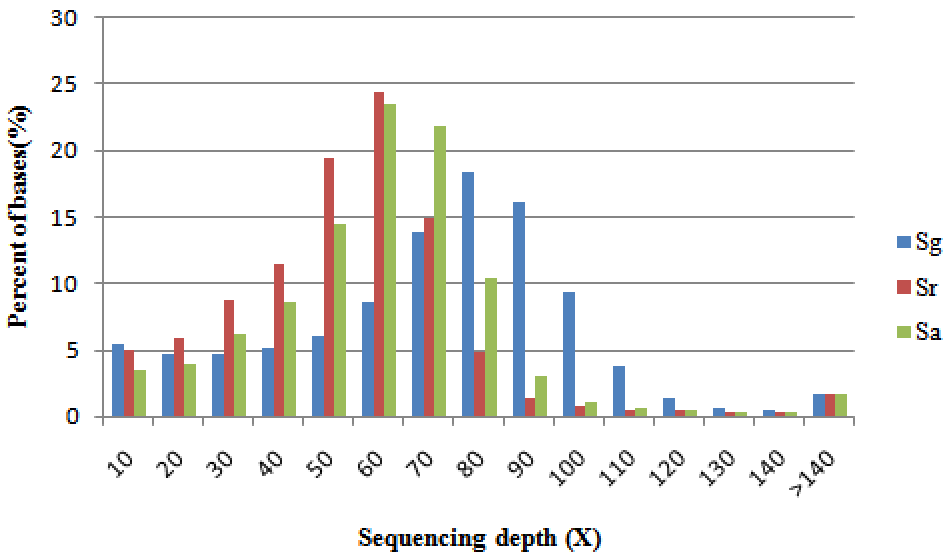

Figure S5. Sequencing depth of the three *Sinocyclocheilus* species. The filtered reads were aligned onto the assembled genome sequences using SOAPaligner for calculation of the percentage of bases with different depth frequency in the genomes.

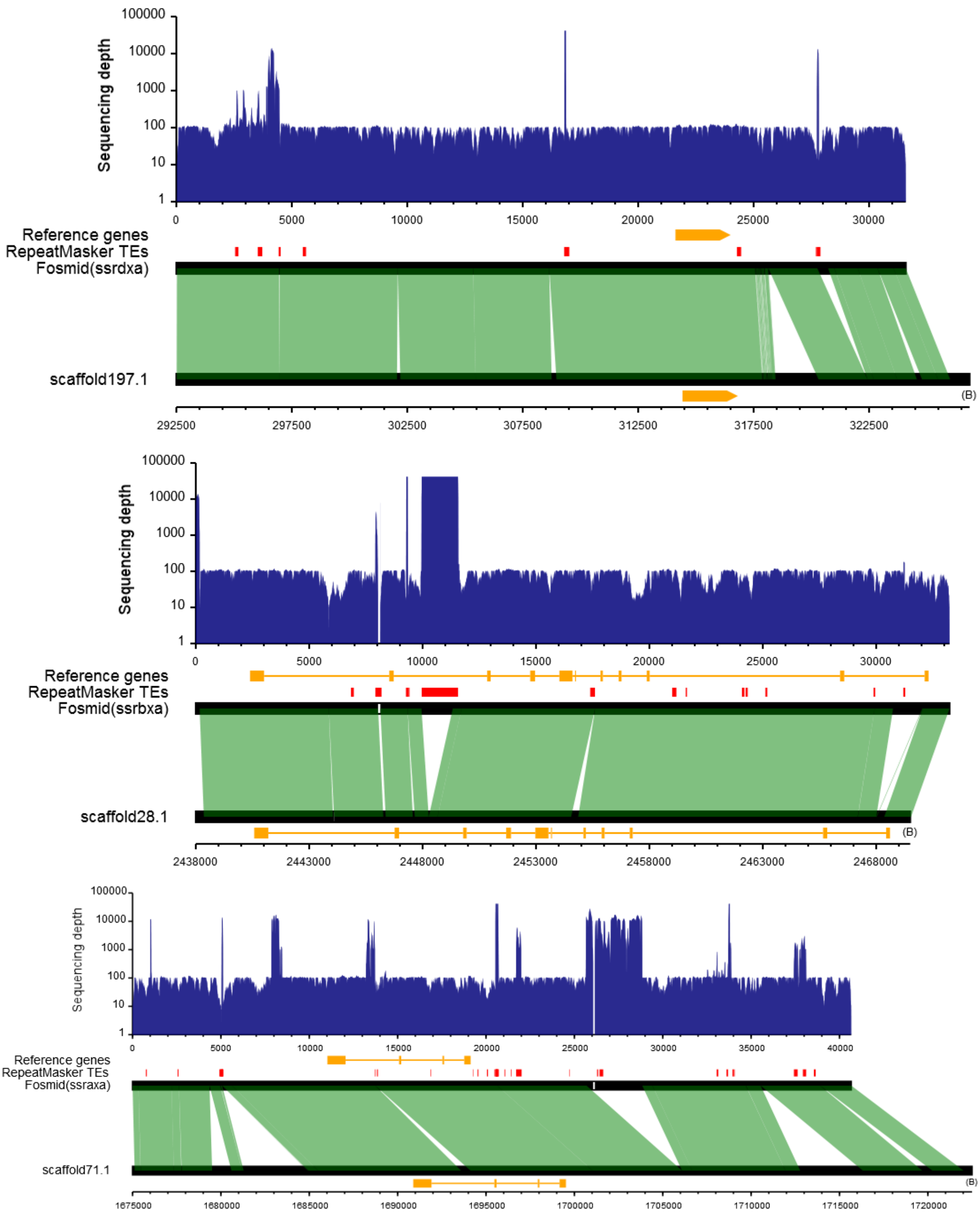

Figure S6. Comparison of the assembled Sg genome with 3 fosmid sequences. Sequencing depth of the fosmids was calculated by mapping the Illumina short reads onto the fosmid sequences. Here, we performed single-end mapping and reported all the repetitive hits. The predicted genes and annotated TEs on the fosmid sequences are shown in orange and red respectively. The remaining unclosed gaps on the scaffolds and fosmid sequences are marked as white blocks. Note that most of the gaps are located in repeat regions, especially in recently evolved repeats with high sequencing depth.

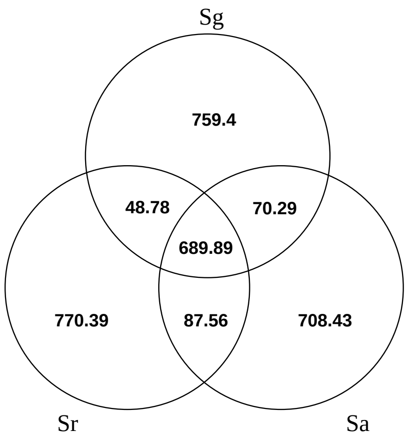

Figure S7. Conserved sequences (Mb) among the three *Sinocyclocheilus* genomes.
