# Supplementary material for: The Sinocyclocheilus cavefish genome provides insights into cave adaptation
Source: BMC Biol. 2016 Jan 4;14:1. doi: 10.1186/s12915-015-0223-4 (PMC4698820; doi:10.1186/s12915-015-0223-4)
Supplement: Additional file 4: Figure S8–S14. — The characteristics of repeats and genes, including the distributions of TEs (Figure S8), mRNA, CDS, exon and intron lengths (Figure S9), double-conserved synteny between Dr and Sg genomes (Figure S10), the evolution of Hox genes (Figure S11) and the similarity analysis (Figure S12), protein orthology comparison (Figure S13), and shared orthologous groups among Dr and three Sinocyclocheilus genomes (Figure S14). (PDF 666 kb) [file 12915_2015_223_MOESM4_ESM.pdf]

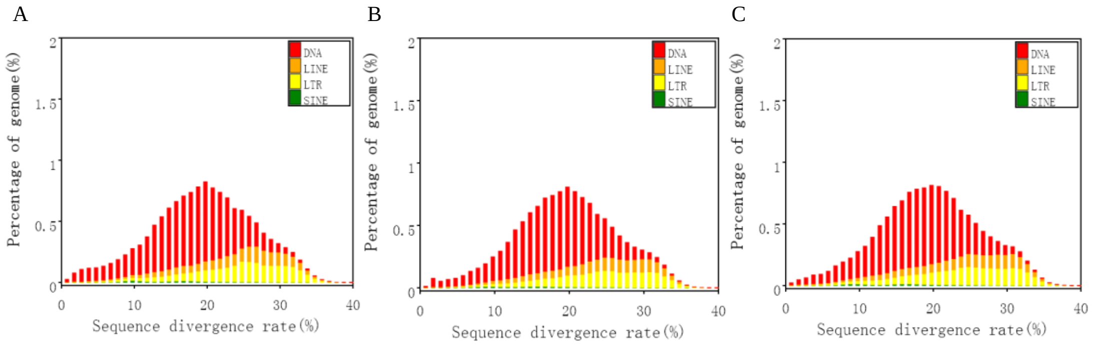

Figure S8. Distribution of divergence rate for each type of TE in the three *Sinocyclocheilus* genomes. (a) Sg, (b) Sr, and (c) Sa. The divergence rate was calculated between the identified TE elements in the genomes by homology-based method and the consensus sequence in the Repbase. The substitute rate distribution shows a peak at 20%, indicating that a large number of copies were inserted 57 MYA ago (the rate of substitution site per year was 3.51×10^{-9}).

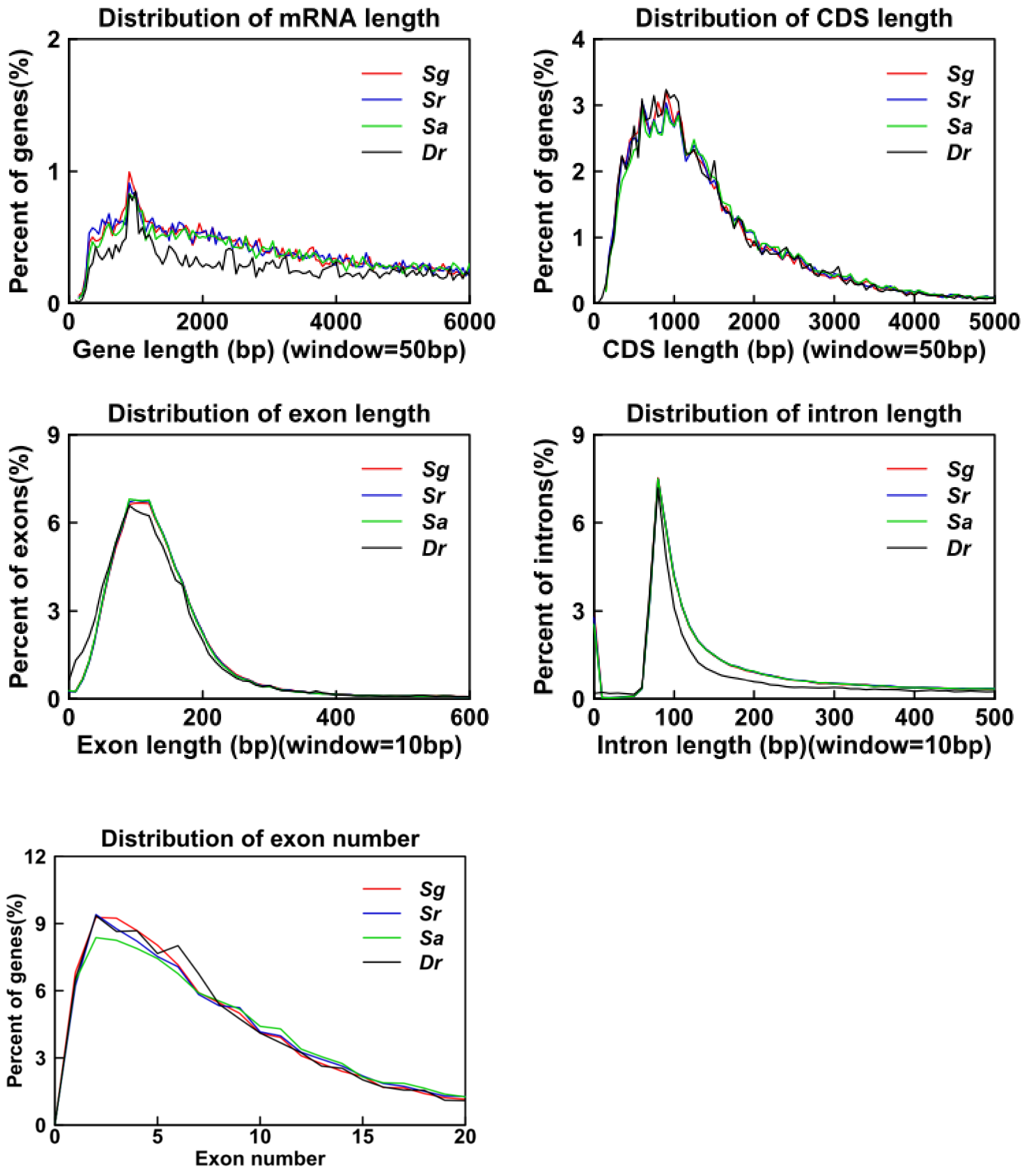

Figure S9. The distributions of mRNA, CDS, exon and intron lengths in Ensembl annotated protein-coding genes of four genomes. Dr: zebrafish (*Danio rerio*).

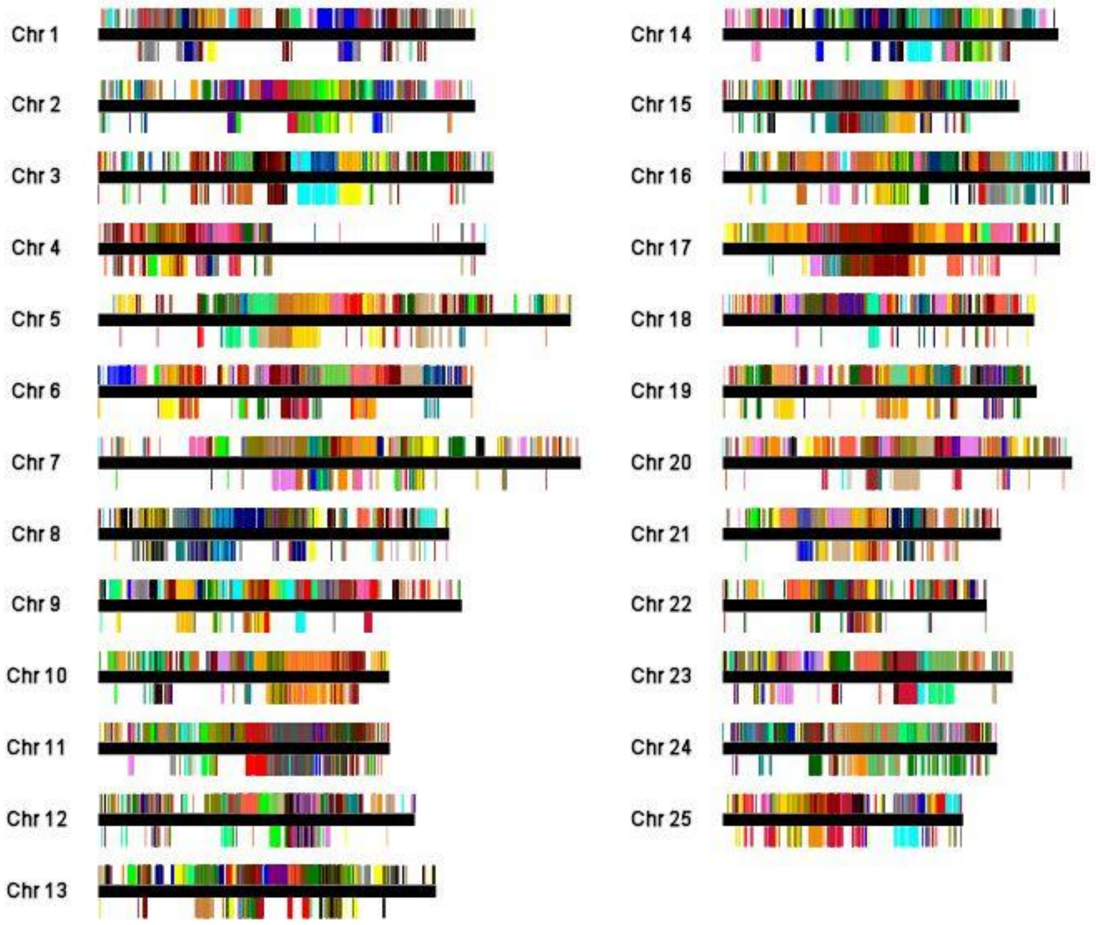

Figure S10. Double-conserved synteny between Dr and Sg genomes. The horizontal black line represents each Dr chromosome, syntenic Sg regions are marked on either side by different colors which represent different scaffolds. Only genes anchored to a Dr chromosome are painted. The data also support one more round of genome duplication in the Sg (compared to the Dr).

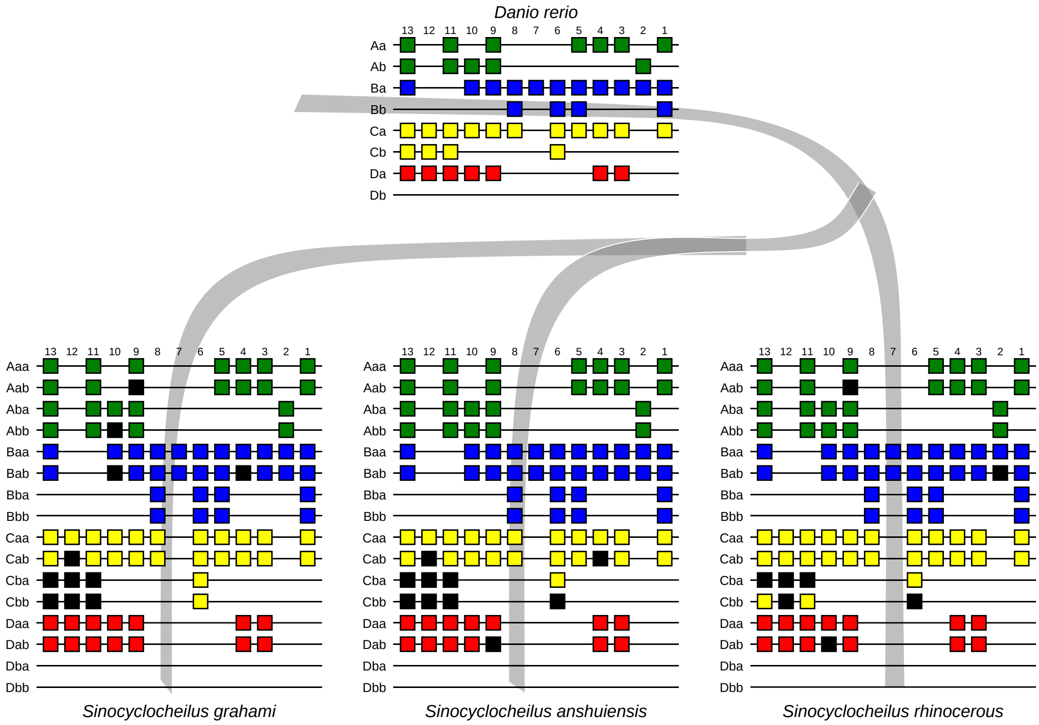

Figure S11. A cladistic model for the evolution of vertebrate Hox clusters from Dr to the three *Sinocyclocheilus* species. Horizontal lines indicate each Hox cluster. Boxes with different colors indicate different *Hox* genes, and black boxes represent the *Hox* genes which were lost.

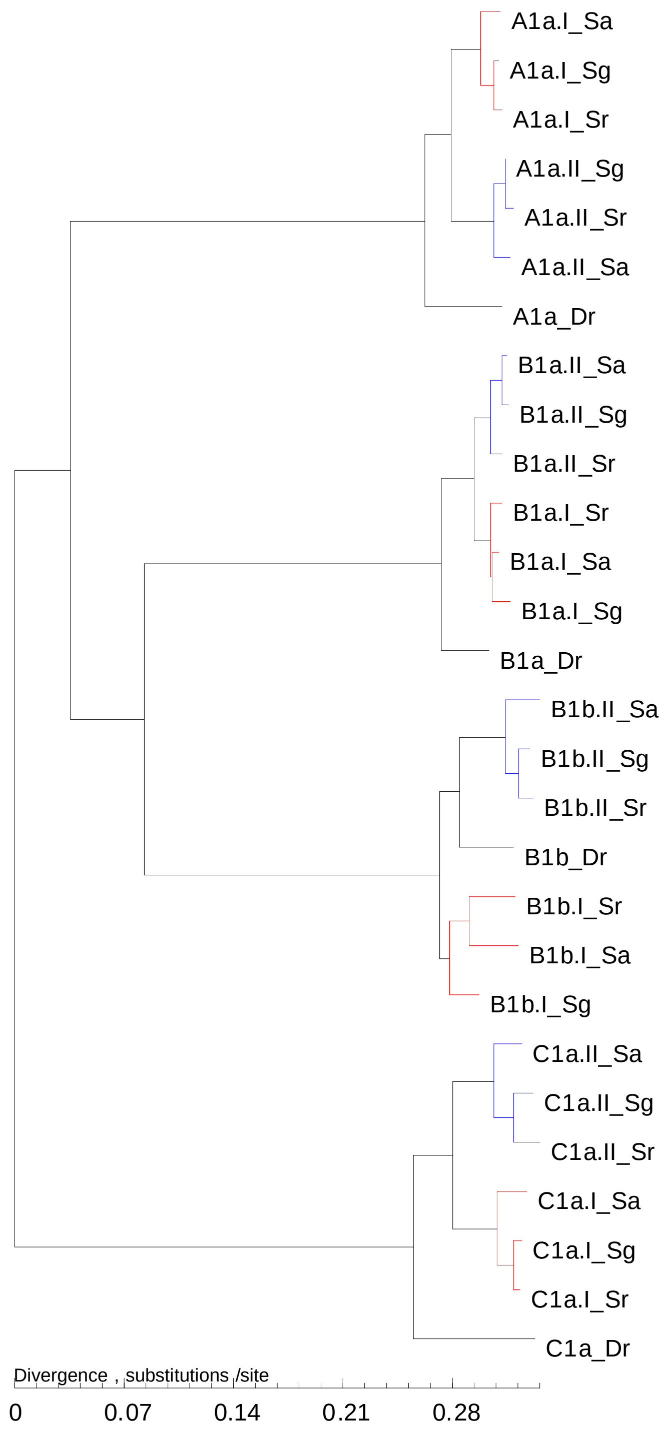

Figure S12A. Similarity analysis of Hox genes. The network was constructed from amino acid sequences of group 1 genes with NJ of TreeBeST showed that Dr had one copy but the *Sinocyclocheilus* species (Sg, Sr and Sa) had two copies (I and II) of HoxA, HoxB and HoxC clusters. Interestingly, Type I and II of each Hox cluster in the *Sinocyclocheilus* fishes were separated groups while Dr was the basic lineage, suggesting that genome duplication occurred before the divergence of Sg, Sr and Sa lineages.

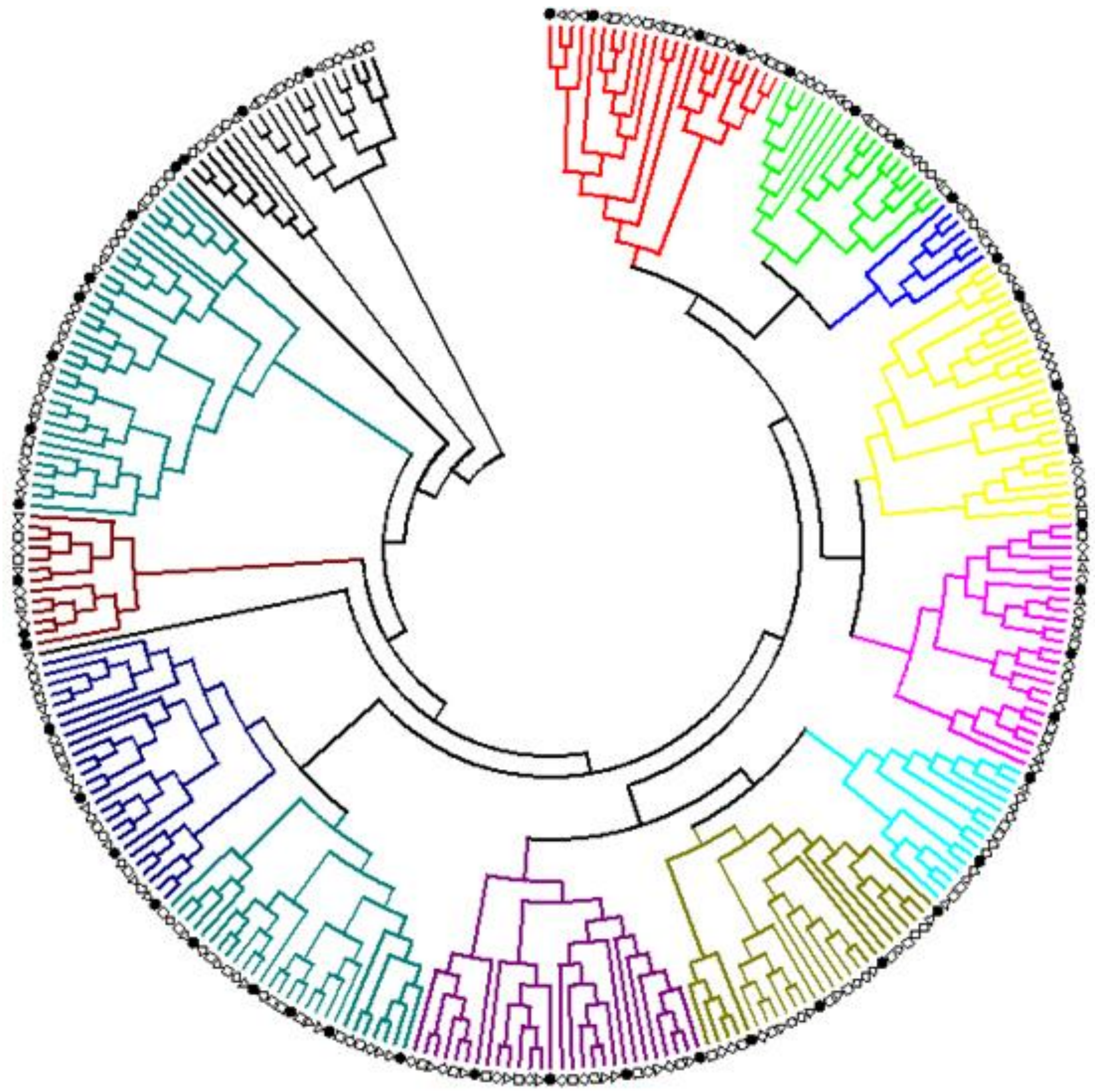

Figure S12B. Similarity cluster of Hox genes from the three *Sinocyclocheilus* species and Dr. □ for Sg, ◇ for Sr, △ for Sa and ● for Dr. The network was constructed from amino acid sequences with NJ of TreeBeST.

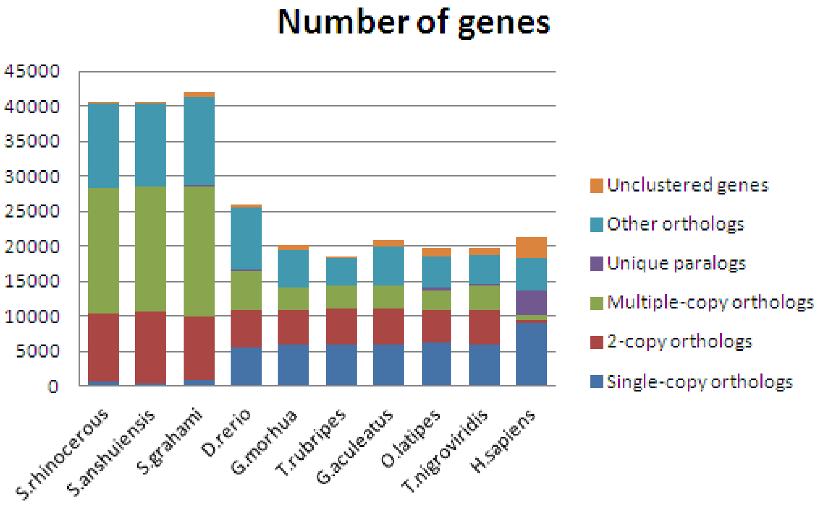

Figure S13. Protein orthology comparison among ten sequenced vertebrate genomes. The ten species include Sg, *Sr*, Sa, Dr, Atlantic cod (*G. morhua*), fugu (*T. rubripes*), three-spined stickleback (*G. aculeatus*), medaka (*O. latipes*), green spotted puffer (*T. nigroviridis*) and human (*H. sapiens*). Single-copy orthologs represent one copy genes in each species. Two-copy orthologs means two copies in each species. Multiple-copy orthologs represent genes with multiple copies.

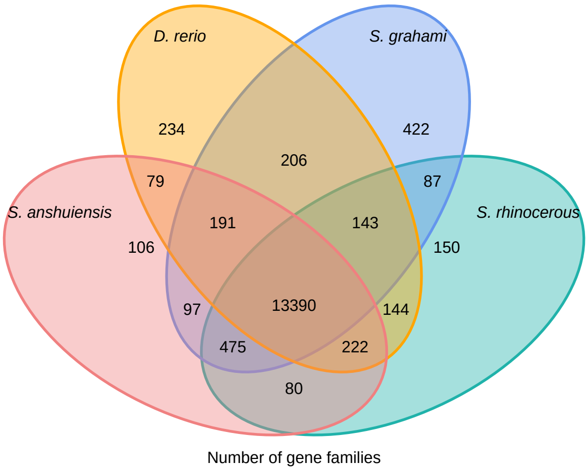

Figure S14. Venn diagram showing shared orthologous groups among four fish genomes.

The four fishes include Dr and the three *Sinocyclocheilus* species.
