# Supplementary material for: The Sinocyclocheilus cavefish genome provides insights into cave adaptation
Source: BMC Biol. 2016 Jan 4;14:1. doi: 10.1186/s12915-015-0223-4 (PMC4698820; doi:10.1186/s12915-015-0223-4)
Supplement: Additional file 5: Figure S15–S17. — The phylogenetic trees and divergence time analyses, including the phylogenetic trees based on CDS, protein, 1st, 2nd, 1st and 2nd, 3rd, 4d codon position of codon sequences of 3,181 genes (Figure S15), mitochondrial CDS sequences (Figure S16), and divergence times calibrated by five fossil records (Figure S17). (PDF 170 kb) [file 12915_2015_223_MOESM5_ESM.pdf]

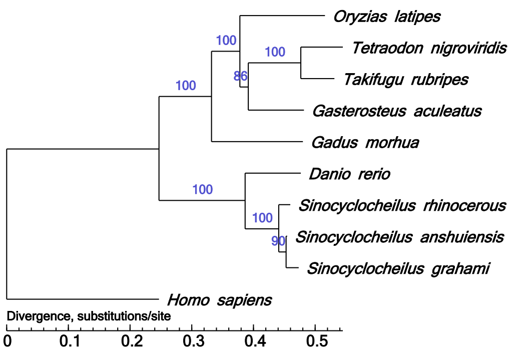

ML, CDS, phash 0

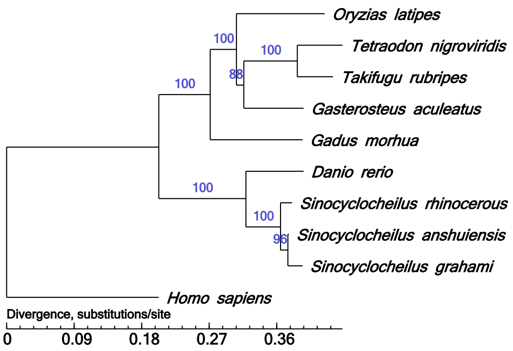

ML, CDS, phash 01

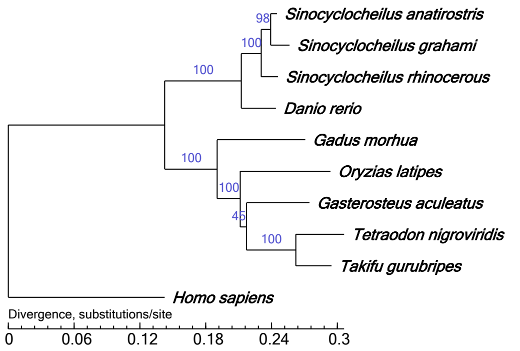

ML, CDS, phash 1

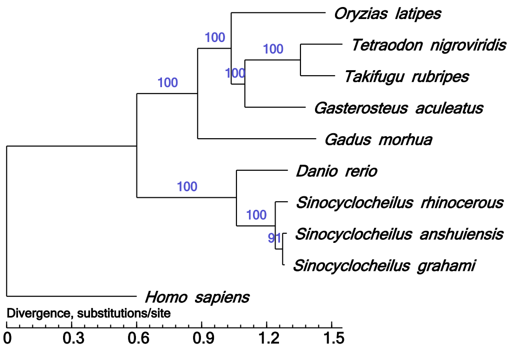

ML, CDS, phash 2

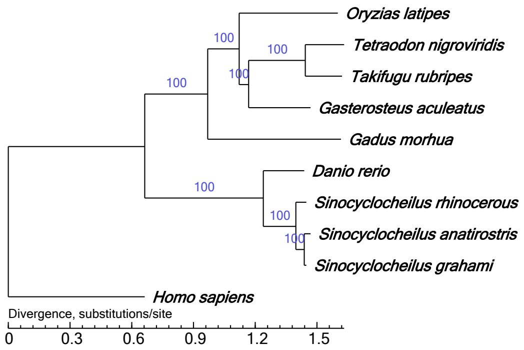

ML, CDS, 4d

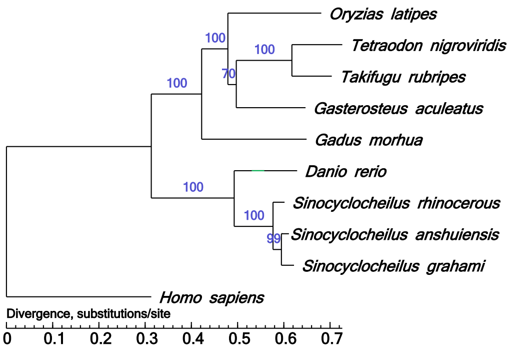

ML, pep, all

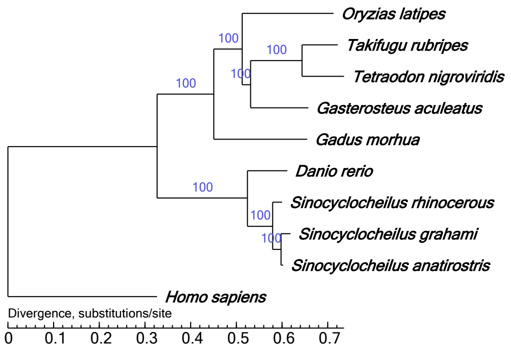

Bays, cds, all

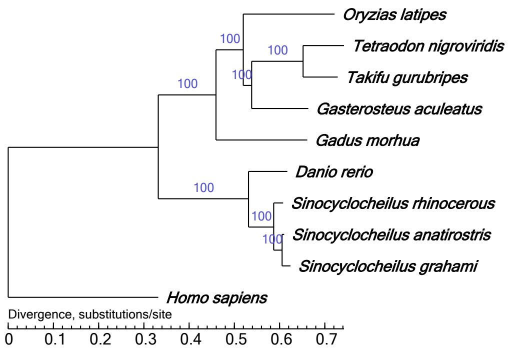

ML, cds, all

Figure S15. Phylogenetic tree of 10 sequenced vertebrates. The tree was constructed with PhyML under GTR/JTT+gamma model based on CDS, protein, 1st , 2nd , 1st& 2nd , 3rd , 4d codon position of codon sequences in 3,181 genes. Each tree was run with 1,000 replications. All internal branches of the tree were 100% bootstrap supported. Note that all the tree

topologies are similar, supporting that Sg is closer to Sa.

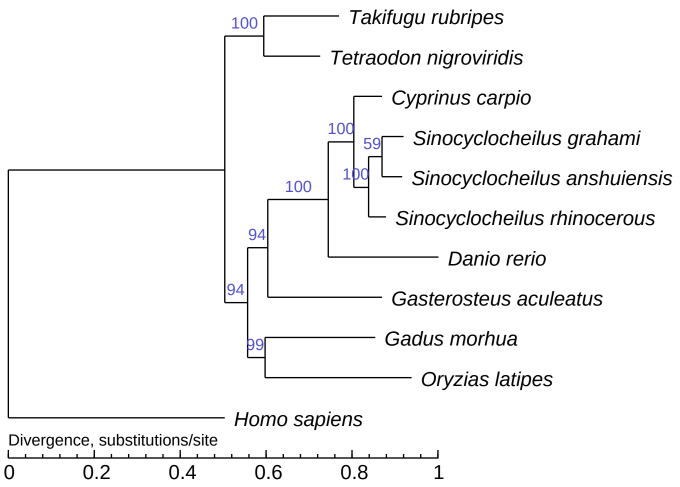

Figure S16. Phylogenetic tree based on mitochondrial CDS sequences of 10 vertebrate species with Bayes analysis.

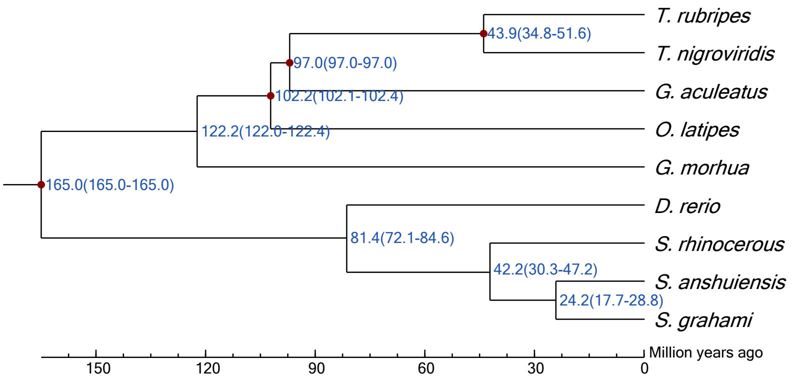

Multidivtime, cds

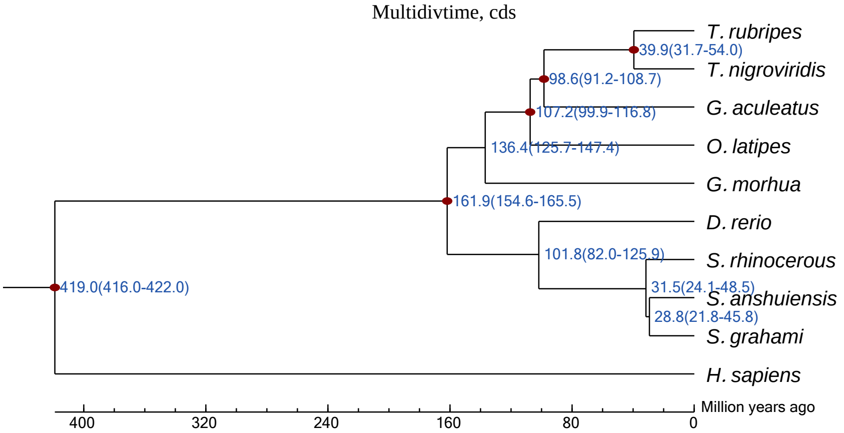

MCMC, phase01

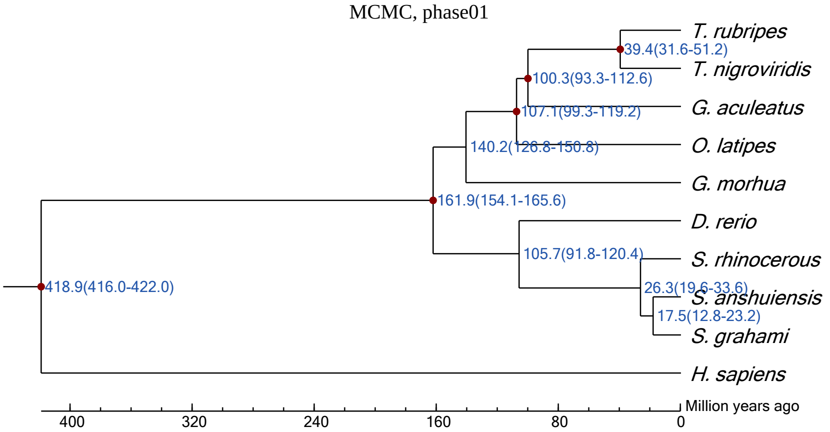

MCMC, 4d

Figure S17. Divergence time of 10 sequenced vertebrates. The divergence time was estimated by PAML mcmctree or Multidivtime based on 1st& 2nd, CDS, and 4d codon position, respectively. The red dots indicated the reference divergence times from the TimeTree (http://www.timetree.org/).
