# Supplementary material for: The Sinocyclocheilus cavefish genome provides insights into cave adaptation
Source: BMC Biol. 2016 Jan 4;14:1. doi: 10.1186/s12915-015-0223-4 (PMC4698820; doi:10.1186/s12915-015-0223-4)
Supplement: Additional file 6: Figure S18–S28. — Analyses to the specific genes, including Tyr (Figure S18), Ush2a (Figure S19), Edar (Figure S20), Oca2 (Figure S21), Mpv17 (Figure S22), Rom1b (Figure S23), Skp1 (Figure S24), and differential expression of eye-related genes, transcriptional factors (Figure S25) and circadian rhythm pathway genes (Figure S26), and similarity cluster of the Tlr gene family (Figure S27) and crystallins (Figure S28). (PDF 1295 kb) [file 12915_2015_223_MOESM6_ESM.pdf]

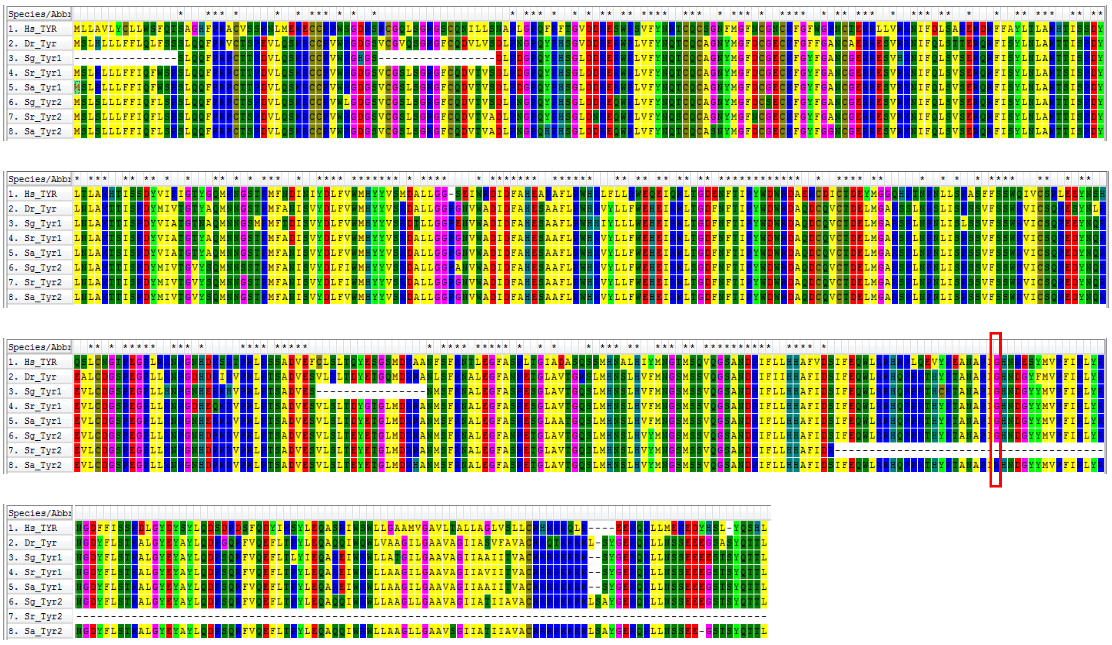

Figure S18. Tyr protein alignment. The Tyr protein from human (Hs), Dr, Sg, Sr, Sa were aligned by MUSCLE. Two copies of *Tyr* genes were found in *Sinocyclocheilus*, encoding proteins named as Tyr1 and Tyr2. Three small deletions were identified in Sg Tyr1, and the premature termination was occurred in Sr Tyr2. Besides that, a G420R mutation was identified in Sa Tyr2 (shown in red rectangle), which was also reported in the Caucasian patients. This mutation site located at exon IV, and previous studies reported that a cluster including this site could be an important functional domain in thse Tyr.

A

```
Human   EILEVFSGDLLRLHAQSHCRCPGSHPRVHPLAQRYCIPNDAGDTADNRVSRLNPEAHPLS
Sg      EIEEVYTGQFPHLHTQSTCRCPASHPKVHPLVERYCIPNAANDTTHNKVLRLNQDAHPLH
Sr      EIEEVYTGQFPHLHTQSTCRCPASHPKVHPLVERYCIPNAANDTTHNKVLRLNQDAHPLH
Sa      EIEEVYTGQFPH-HTQSTCRCPASHPRVHPLVERYCIPNAANDTTHNKVLSLNQDAHPLH
```

B

```
Human   FVNDNDVGTSWVSNVFTNITQLNQGVTISVDLENGQYQVFYIIIQFFSPQPTEIRIQRKK
Sg      YINDNDIGTTWISSIFPTLELLDKGITISIDLENGQYQVFYVILQFLNAQPEAVHIQRKT
Sr      YINDNDIGTTWISSIFPTLELLDKGITITIDLENGQYQVFYVILQFLNAQPEAVHIQRKT
Sa      YINDNDIGTTWNSSVFPTLELLDKGITITIDLENGQYQAFYVILQEYA------------
```

C

```
Human   NQCEPHRYNLTIDNFQH-CQMCECDSLGTLPGTICDPISGQCLCVPNRQGRRCNQCQPGF
Sg      NRCSSHTYNLSSANATHGCQPCHCDHMGTVPATVCNPVSGQCICLPTRYGQDCGTCKPGF
Sr      NRCSSHTYNLSSANATHGCQPCHCDHMGTVPATVCNPVSGQCVCLPTRNGQDCGTCKPGF
Sa      NRCSSHTYNLSSANAMHGCQPCHCDHMGTVPATVCDPVSGQCFCLPTRYDXDCGTCKPGF
```

D

```
Human   LRPPLVKGINSTTIHLRWFPPEELNGPSPIYQLERRESSLPALMTTMMKGIRFIGNGYCK
Sg      QSAPTLRPLNMTVMKVSWDAPAELNGPPPLYHVERTDVSLSDTQGQVIRGRRFTGSGYFR
Sr      LSAPTLRPLNMTVMKVSWDAPAELNGPPPLYHVERTDVSLSDTQGQVIRGRRFTGNGYFR
Sa      LRAPALRPLNMTTMKVSWDASAELNGPPPLXHVERTDVSLSDAQGQVIRGRRFTGSGYFR
```

Figure S19. Ush2a protein alignment. The Ush2a protein from human, Sg, Sr, Sa were aligned by MUSCLE. The mutations marked by dark colors are (A) R334S, (B) V382A, (C) the first termination codon, and (D) the second termination codon. Previous studies showed that the former two mutation sites may slightly affect splicing, while the existence of several termination codons indicate that Sa *Ush2a* may be a pseudogene.

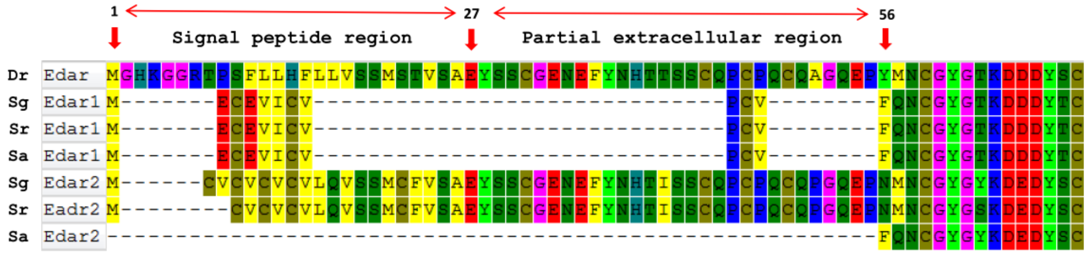

Figure S20. Deletions in Edar (Ectodysplasin-A receptor) of Sa. The Edar protein from Dr, Sg, Sr, Sa were aligned by MUSCLE. Compared with the *Edar*gene in Dr, two copies of *Edar*genes were identified in three *Sinocyclocheilus* species, encoding proteins named Edar1 and Edar2 respectively. There were deletions in the signal peptide region and partial extracellular region in all three Edar1, which may lead to change or loss of function. The structures of Edar2 in Sg and Sr were relatively complete compared with Sa, while the signal peptide region and partial extracellular region were totally deleted in Edar2 of Sa, which may lead to function loss in guiding the Edar protein transfer across the membrane, and subsequently generate few scales at the skin surface of Sa.

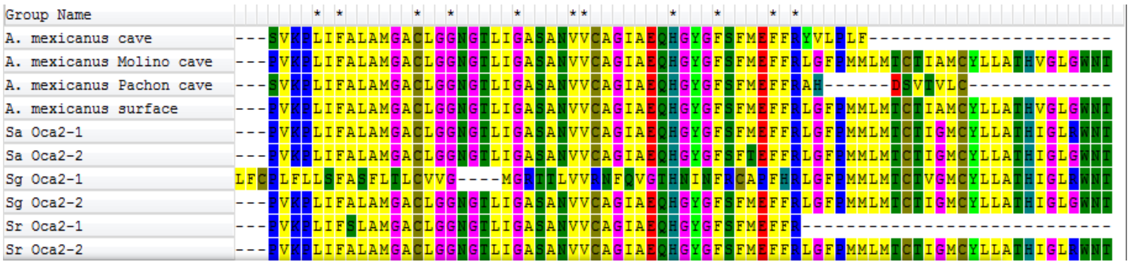

Figure S21. Comparison of Oca2 of *A. mexicanus* cavefish with the three *Sinocyclocheilus* species. The Oca2 protein from *A. mexicanus*, Sg, Sr, Sa were aligned by MUSCLE. The deletions of *A. mexicanus* cavefish Pachon population may produce function loss in pigmentation. No similar results were found in this population and Sa.

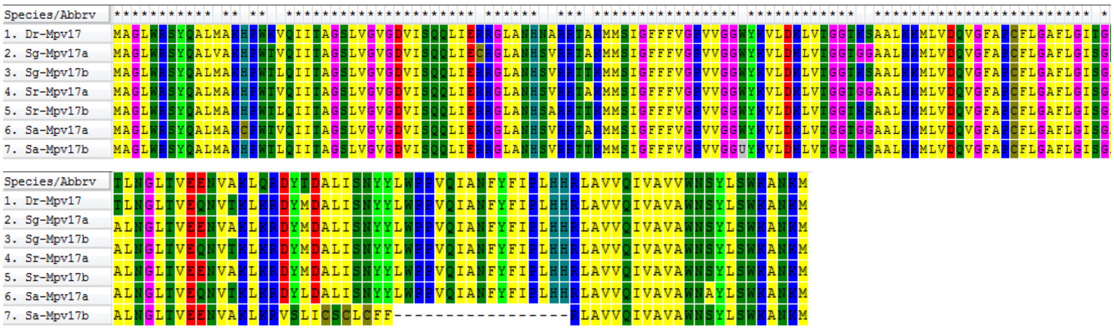

Figure S22. Mutation of Mpv17 protein in Sa. The Mpv17 protein from Dr, Sg, Sr, Sa were aligned by MUSCLE. The mutant and deleted sequence of Sa may lead to functional decline in Mpv17 protein, which may influence listening, or pigment formation.

A.

B.

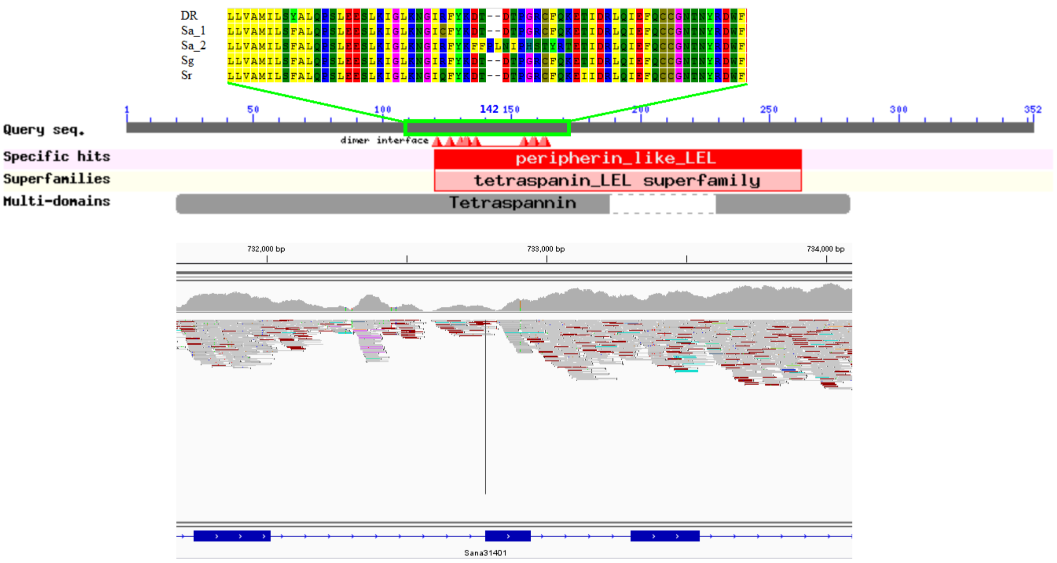

Figure S23. A 2-amino-acid insertion in the Sa Rom1b protein. (A) The structural variation in the Rom1b protein was compared with those in Dr and two *Sinocyclocheilus*species (Sg and Sr). The inserted site is located in Tetraspanin family conserved domain [NCBI accession: pfam00335]. (B) IGV (Integrative Genomics Viewer) shows the breaking point of sequencing depth nearby the insertion site.

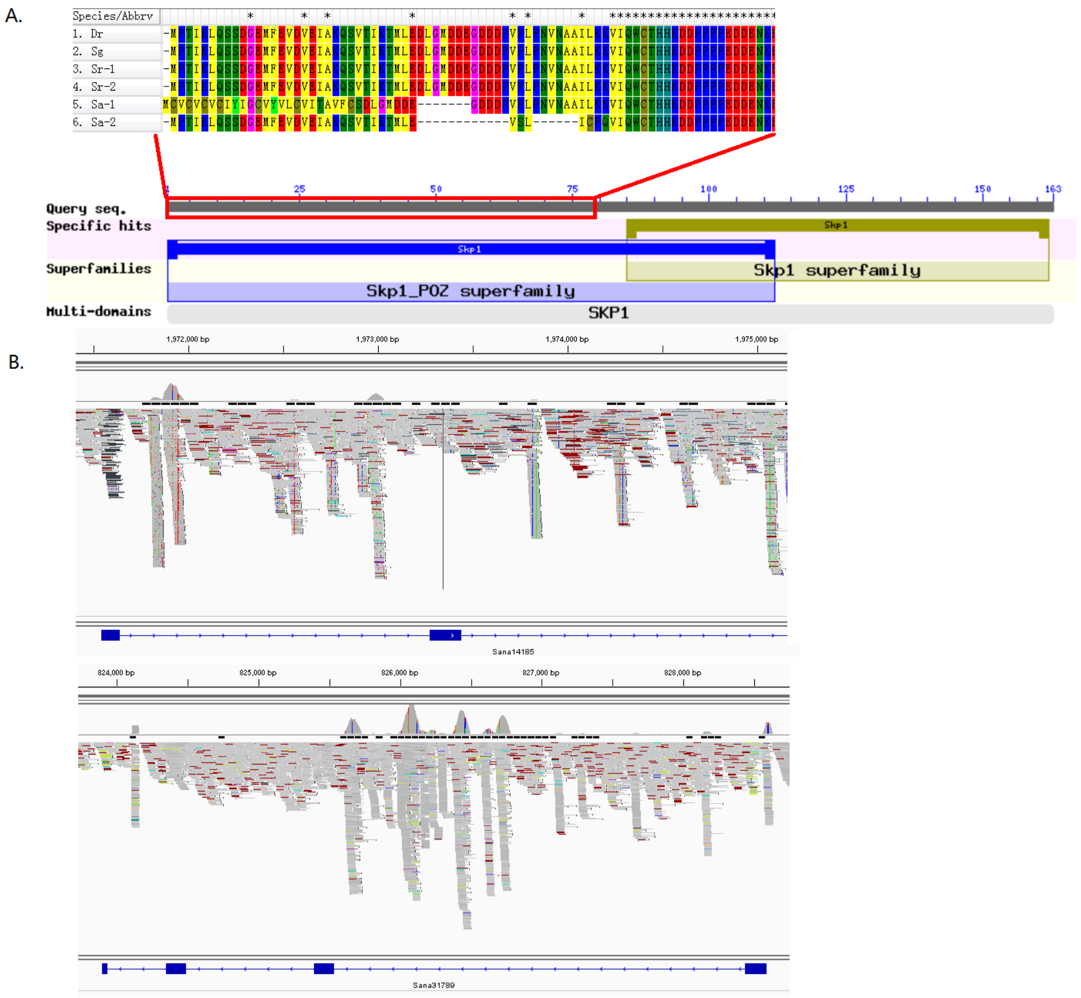

Figure S24. Structural variation in the Skp1 protein. (A) Comparison among Dr and the sequenced *Sinocyclocheilus* species reveals the Skp1 structural variation in Sa. The N-terminal deletions of Sa Skp1proteins (Sa-1 and Sa-2) are located in the Skp1_Poz superfamily conserve domain. (B) IGV (Integrative Genomics Viewer) shows the breaking point of sequencing depth near the deletion sites.

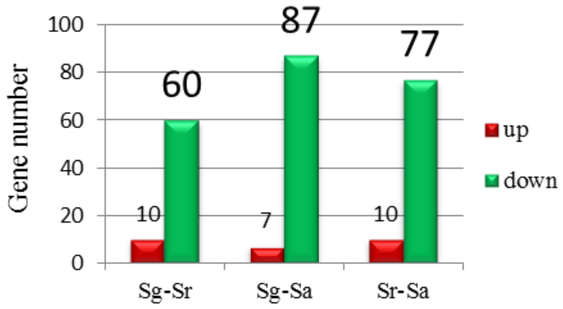

Figure S25A. Differential expression of eye-related genes in the three *Sinocyclocheilus* species among the 140 examined genes. All of the differential expression statuses were tested with FDR less than 0.01.The data demonstrate that the status of the transcriptional factors' expression declines as Sg>Sr>Sa. See more details in Table S26.

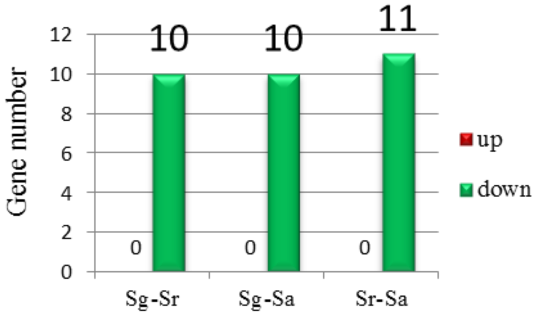

Figure S25B. Differential expression of eye development related transcriptional factors in the three *Sinocyclocheilus* species. Within the 12 analyzed transcriptional factors, compared with Sg, both Sr and Sa have 10 down-regulated (FDR≤0.01) factors; compared with Sr, Sa has 11 down-regulated (FDR≤0.01) factors. In general, the status of the transcriptional factors' expression declines as: Sg>Sr>Sa. See more details in Table S26.

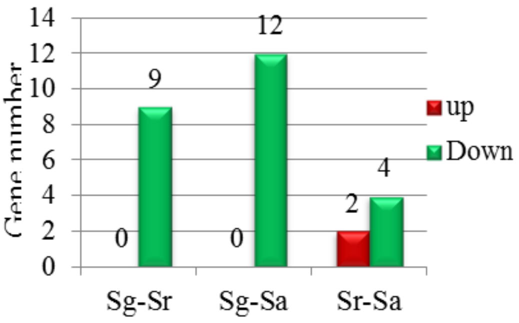

Figure S26. Differential expression of circadian rhythm pathway genes. Among the 17 examined genes, compared with Sg, Sr has 9 down-regulated genes, while Sa has 12 down-regulated genes. Compared with Sr, Sa has 4 down-regulated genes, but only 2 up-regulated genes. All of the differential expression statuses were tested with FDR less than 0.01. Generally speaking, the status of the rhythm pathway genes' expression declines as Sg>Sr>Sa, implying that the cavefish have no or low circadian rhythms.

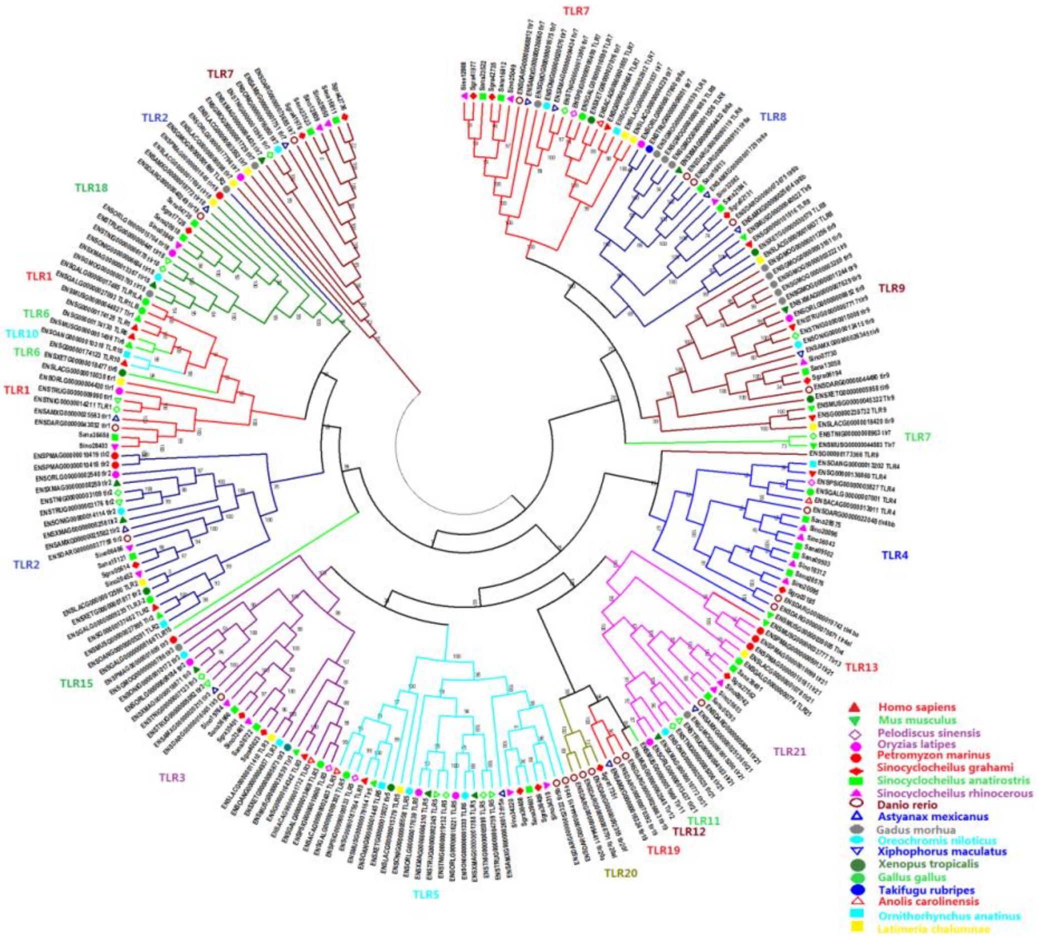

Figure S27. Similarity cluster of the *Tlr* gene family. The *Sinocyclocheilus Tlr* genes are closer to those of Dr. Each category is marked in a different color.

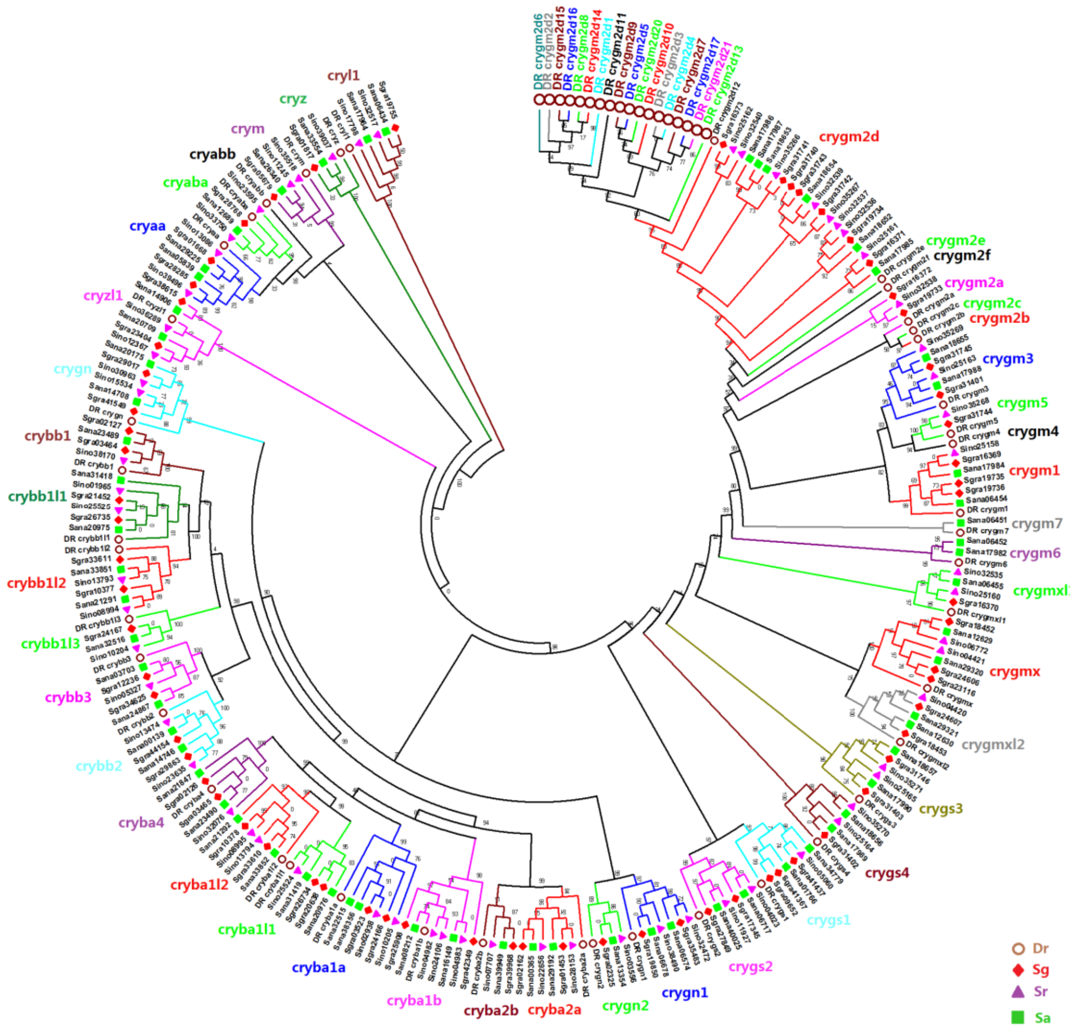

Figure S28. Similarity cluster of crystallins among Dr and the three *Sinocyclocheilus* species. The *Sinocyclocheilus* genes are classified referring to the Dr gene nomenclature. Each branch is marked by a different color. Among the 60 examined crystallin genes, 29 Dr genes are defined as outgroups compared to the *Sinocyclocheilus* genes, and 4 Dr genes cannot be distinguished from those in the *Sinocyclocheilus* species.
