# Supplementary material for: The Sinocyclocheilus cavefish genome provides insights into cave adaptation
Source: BMC Biol. 2016 Jan 4;14:1. doi: 10.1186/s12915-015-0223-4 (PMC4698820; doi:10.1186/s12915-015-0223-4)
Supplement: Additional file 7: Figure S29–S32. — Morphological analyses of the three Sinocyclocheilus species, including scales (Figure S29), swim bladder (Figure S30), lateral line system (Figure S31) and taste buds (Figure S32). (PDF 434 kb) [file 12915_2015_223_MOESM7_ESM.pdf]

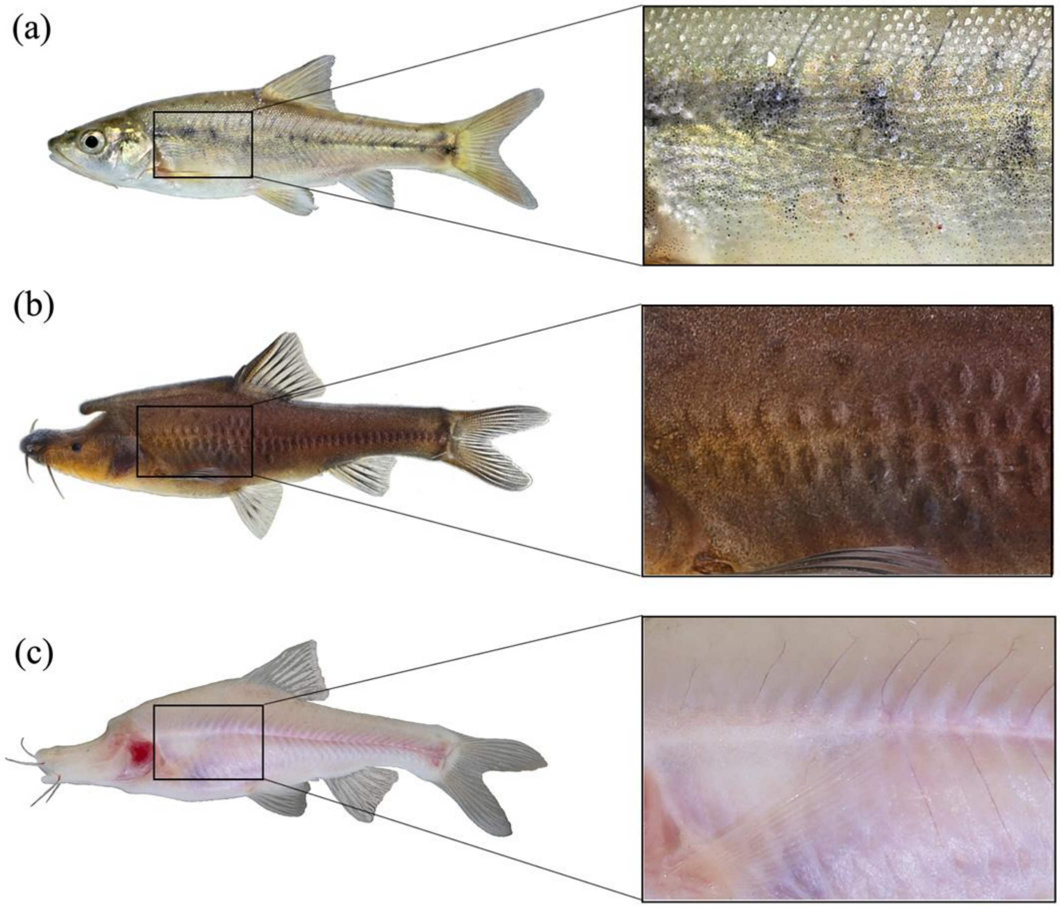

Figure S29. Scale coverings of the three *Sinocyclocheilus* species. The photos on the right show the anterior trunk scales including lateral line scales. Sg shows normal trunk scales and expanded lateral line scales; Sr shows rudimental trunk scales and expanded lateral line scales; and Sa shows rudimental trunk scales and lateral line scales.

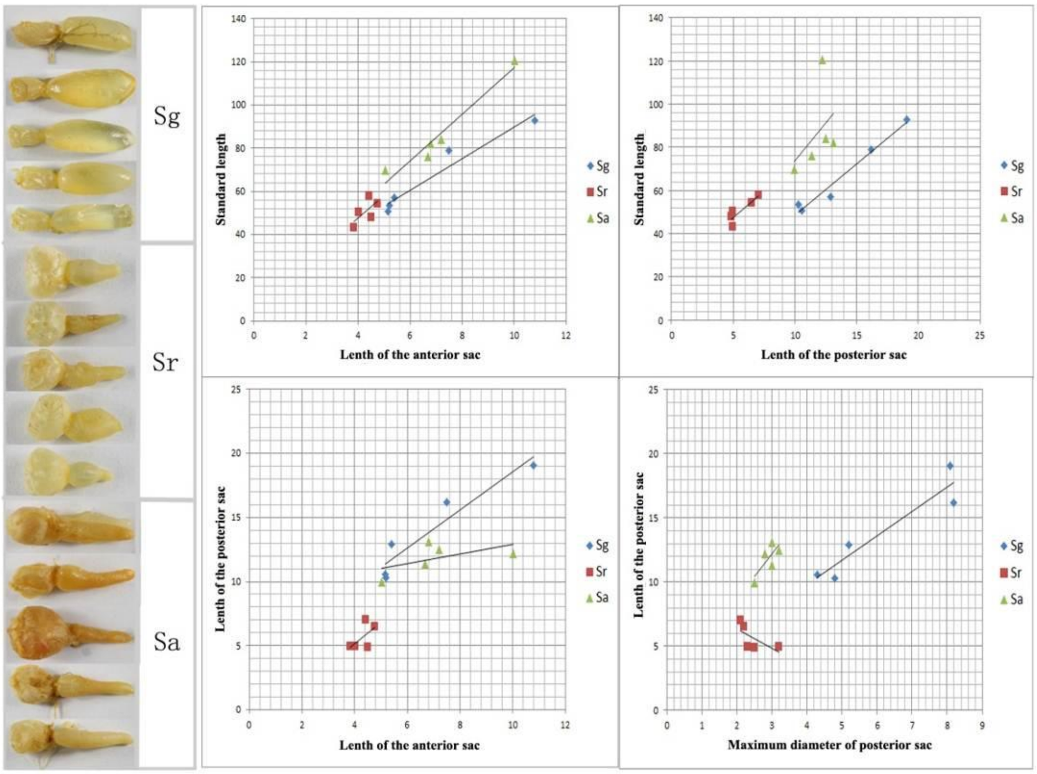

Figure S30. Morphology of the swim bladder of the three *Sinocyclocheilus* species. The photos on the left show the soft texture of the swim bladder in Sg and Sr, while it is somewhat harder in Sa. The posterior sac of the cavefish Sa has degenerated to a long tube. The data are provided in the scatter plots on the right.

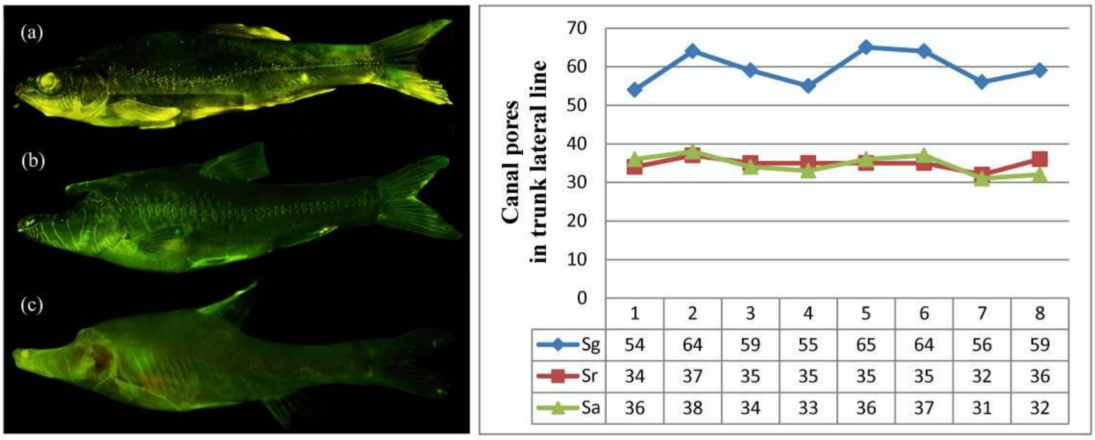

Figure S31. Trunk lateral line system in the three *Sinocyclocheilus* species. The photos on the left show distributions of neuromasts in the body surface (including trunk lateral line) after DASPEI staining of (a) Sg, (b) Sr and (c)Sa. The line chart in the right shows the number of lateral scales in the adult fishes.

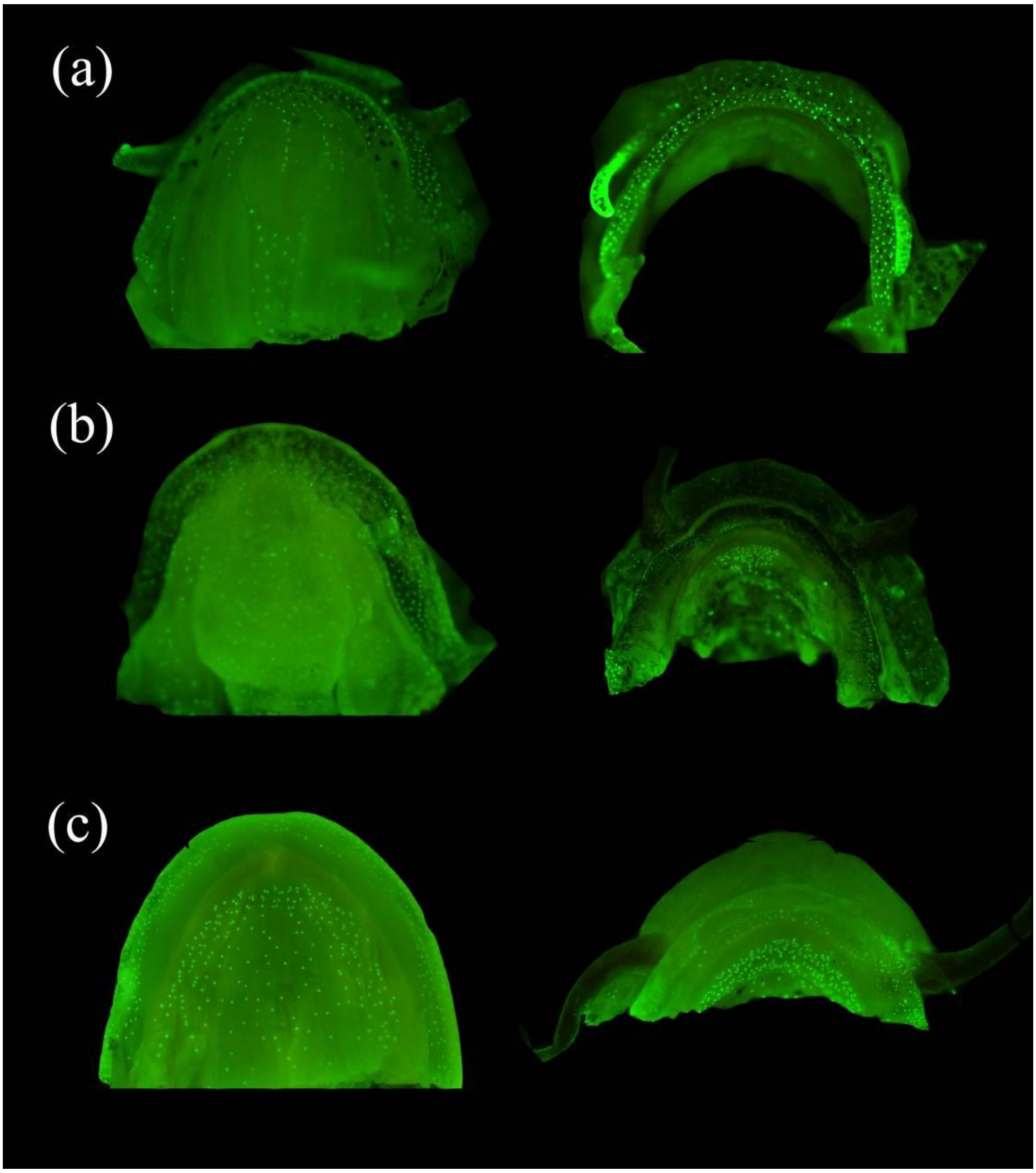

Figure S32. Taste buds on the outer lower jaw (left) and inner upper jaw (right) of (a) Sg, (b) Sr and (c) Sa. This figure shows that taste bud density within the jaw of the adult fishes increases in the following order: Sg<Sr<Sa.
