# Supplementary material for: The Sinocyclocheilus cavefish genome provides insights into cave adaptation
Source: BMC Biol. 2016 Jan 4;14:1. doi: 10.1186/s12915-015-0223-4 (PMC4698820; doi:10.1186/s12915-015-0223-4)
Supplement: Additional file 9: — Notes S1–S6. The supplementary notes to the article, including the organism background, genome sequencing and assembly (Note S1), genome annotation (Note S2), transcriptome analysis (Note S3), evolutionary analyses (Note S4), morphological comparison analysis (Note S5), and cave adaption analysis in the aspects of vision, pigmentation, scale development, hearing, immune response, circadian rhythm and sense of taste (Note S6). (PDF 668 kb) [file 12915_2015_223_MOESM9_ESM.pdf]

Additional Notes for

The *Sinocyclocheilus* cavefish genome provides insights into cave adaptation

Junxing Yang*†, Xiaoli Chen †, Jie Bai†, Dongming Fang†, Ying Qiu†, Wansheng Jiang†, Hui Yuan, Chao Bian, Jiang Lu, Shiyang He, Xiaofu Pan, Yaolei Zhang, Xiaoai Wang, Xinxin You, Yongsi Wang, Ying Sun, Danqing Mao, Yong Liu, Guangyi Fan, He Zhang, Xiaoyong Chen, Xinhui Zhang, Lanping Zheng, Jintu Wang, Le Cheng, Jieming Chen, Zhiqiang Ruan, Jia Li, Hui Yu, Chao Peng, Xingyu Ma, Junmin Xu, You He, Zhengfeng Xu, Pao Xu, Jian Wang, Huanming Yang, Jun Wang, Tony Whitten*, Xun Xu*, Qiong Shi*

† These authors contributed equally to this work.

* To whom correspondence should be addressed. E-mail: yangjx@mail.kiz.ac.cn (JY), Tony.Whitten@fauna-flora.org (TW), xuxun@genomics.cn (XX), or shiqiong@genomics.cn (QS)

Contents

Note S1 3
1. Organism background, genome sequencing and assembly 3
1.1 Organism background 3
1.2 Sample background and sequencing 3
1.3 Genome size estimation by k-mer analysis and genome assembly 3
1.4 Assessment of genome assemblies 4
Note S2 6
2. Genome annotation 6
2.1 Repetitive sequence detection 6
2.2 Protein-coding gene annotation 6
2.3 Non-coding RNA annotation 7
Note S3 9
3. Transcriptome analysis 9
3.1 RNA isolation, and RNA-Seq method 9
3.2 RNA-Seq data processing 9
3.3 Alignment and *de novo* assembly of the transcriptomes 9
Note S4 10
4. Evolutionary analysis of *Sinocyclocheilus* genomes 10
4.1 Gene family cluster 10
4.2 Phylogenetic analysis 10
4.3 Divergence time estimation 11
4.4 Demographic history 11
4.5 Gene family contraction and expansion 11
4.6 Genes with accelerated evolutionary rates 12
4.7 Evolution of Hox clusters 13
4.8 Loss of Sa-specific gene families 14
Note S5 16
5. Morphological comparison analysis 16
5.1 Paraffin section of eyes of the three *Sinocyclocheilus* species 16
5.2 Immunocytochemistry of taste buds 16
5.3 Anatomy of gas bladder, absolute fecundity and other measurements 17
5.4 Synchrotron X-ray microtomography of the saccular otolith 17
Note S6 18
6. Cave adaption analysis 18
6.1 Vision analysis 18
6.2 Pigmentation 19
6.3 Scale development 20
6.4 Hearing 20
6.5 Immune response 21
6.6 Circadian rhythm 21
6.7 Sense of taste 22
References (1-85) 23

Note S1

1. Organism background, genome sequencing and assembly

1.1 Organism background

The cyprinid genus *Sinocyclocheilus* (Cypriniformes: Cyprinidae) is endemic to the massive southwestern karst area which abuts the eastern Qinghai-Tibetan Plateau of China, covering the Yunnan-Guizhou Plateau and the surrounding region (including east Yunnan Province, south-central Guizhou Province, and northwest Guangxi Province). There are about 50 valid species distributed within a relatively narrow area (about 270,000 km^2), making it the most species-rich cyprinid genus in China [1]. *Sinocyclocheilus* is well known for its marked adaptations to the subterranean environment. Its high species diversity and phenotypic variation make it an emerging model for studying the evolution of cavefishes.

Three adult female fishes (Sg: *S. grahami*, Sr: *S. rhinocerous* and Sa: *S. anshuiensis*) were selected for whole genome shotgun sequencing in this study, which were collected in Lake Dianchi, Yunnan; Luoping, Yunnan; and Lingyun, Guangxi respectively; representing surface-dwelling, semi-cave-dwelling and cave-restricted species (Figure S1). The research protocol and treatment of experimental animals were reviewed and approved by the internal review board of the Kunming Institute of Zoology, Chinese Academy of Sciences (approval ID: SYDW-2014020).

1.2 Sample background and sequencing

High-quality genomic DNA was extracted from the muscle tissues using Puregene Tissue Core Kit A (Qiagen, Maryland, USA) for construction of libraries with different inserted sizes (250 bp~20 kb) (Table S1). In total, twenty-five paired-end libraries (11 for Sg, 7 for Sr and 7 for Sa, respectively) were generated with the Illumina standard operating procedure. Paired-end sequencing was performed on Illumina Hiseq2000 for each library.

1.3 Genome size estimation by k-mer analysis and genome assembly

There were 293.70, 174.02 and 188.28 Gb raw data obtained for the Sg, Sr and Sa fishes, respectively (Table S1). Artificial and low-quality reads were filtered first and then 88.05, 48.31 and 60.54-fold coverage of *Sinocyclocheilus* genomes were used for assembly (Table S2). We corrected the sequencing errors with 17-mer frequency lower than four with a method described in a previous study [2]. In addition, we estimated genome sizes of the three *Sinocyclocheilus* species using the 17-mer depth frequency distribution method: G (Genome size) =

K-mer_num/Peak_depth. The estimated genome sizes are 1.79, 1.89 and 1.81 Gb, respectively (Figure S3, Table S4). We subsequently assembled the filtered reads into contigs and scaffolds to build the genomes using SOAPdenovo [3] and fulfilled the gaps with GapCloser [3]. Our assembly strategy allowed us to obtain a 1.75-Gb genome for Sg with an scaffold N50=1.16 Mb, a 1.73-Gb genome for Sr with an scaffold N50=896.4 kb and a 1.68- Gb genome for Sa with an scaffold N50=1.25 Mb (Table S3).

To assess the quality of our assemblies, we performed three different evaluations. First, we calculated the GC content of these three fishes (37.3% for Sg, 37.2% for Sr, and 37.3% for Sa), which were similar to those in zebrafish (*Danio rerio*) and medaka (*Oryzias latipes*) (Figure S3). Second, the GC depth distributions were relatively concentrated with a GC content above 20x (Figure S4). Third, we aligned the sequencing reads to the assembled genomes and determined the sequencing depth distribution, which showed that the proportion of sequencing was close to their sequencing depth, and depths lower than 10x were under 5% (Figure S5) [4].

1.4 Assessment of genome assemblies

1.4.1 Evaluation of the Sg assembly with *de novo* assembled fosmids

Three fosmid sequences were also used to check the genome assembly of Sg. Single-end Sanger sequencing was performed, and the average length of generated reads was about 650 bp. After filtering, about 7.5 folds of coverage for each fosmid were used for assembly.

The fosmids were aligned against the scaffolds using BLAT with default parameters. The alignment blocks were then linked on fosmid sequences by in-house Perl scripts. Paired-end reads of short inserted size (< 1000 bp) of Sg were mapped on the fosmid sequences using SOAP (version 2.21). SOAPcoverage (SOAP software package) was applied to calculate sequencing depth for each fosmid sequence with a non-overlapping 100-bp window.

As a result, each fosmid (average length=35 kb) was aligned to only one scaffold (Figure S6), and up to 93.6% of the total fosmid regions (Table S5) were well covered by the assembled contigs.

1.4.2 Assessment of genome assembly coverage

The assessment of genome assembly coverage was carried out using CEGMA program (version 2.3) (http://korflab.ucdavis.edu/datasets/cegma/) [5, 6], which combin í tblastn (blast-2.2.25), genewise (wise2.2.3), hmmer (hmmer-3.0), and geneid (version 1.4). By comparing the completeness of predicted core eukaryotic genes (CEGs) with 248 highly conserved CEGs in the database, we could evaluate the coverage of our assembled genomes.

The results indicated that the ratio of complete CEGs coverage were 79.84%, 81.05% and 85.48% respectively, and the ratios of partial coverage of the three species were more than 96% (Table S9).

1.4.3 Pairwise whole genome alignment

Pairwise whole genome alignment among Sg, Sr and Sa was carried out using LASTZ, with the parameters setting as: C=2, T=2, H=2000, Y=3400, L=6000, and K=2200. Subsequently the Chain/Net package was used for post treatment. The genomes were masked with RepeatMasker repeats at "-s" setting and TRF tandem repeats of period ≤ 12 (Table S6). The aligned length between each two was about 760 Mb (Table S7).

1.4.4 Multiple whole genome alignment

The 3-way whole genome multiple alignment of Sg, Sr and Sa was generated using multiz following the topology of species tree. The Sa genome was set to be the reference, and for input pairwise alignments we carried out in-house generation of the Sg versus Sr and Sg versus Sa alignments (Figure S7).

1.4.5 Detection of segmental duplication in *Sinocyclocheilus* genomes

We analyzed the Sg, Sr and Sa genome assemblies using Whole Genome Analysis Comparison (WGAC) approaches that were designed to detect genomic duplicates >1-kb length and >90% sequence identity.

We applied LASTZ designed for long genomic sequence alignments within Sg, Sr and Sa assemblies. First, self-versus-self LASTZ alignment was performed using the repeat-masked genome sequences, and then chaining of well-ordered neighboring alignments. We obtained the seeding segmental duplications (non-repeat alignment length >500 bp and overall identity >85%), and then reintroduced the masked repeat regions to perform optimal global alignment to refine the alignment identity and define the boundaries of segmental duplications more accurately. The resulting alignments that extended to >1-kb length and had >90% sequence identity were deemed segmental duplications (Table S8).

From the results, we observed that segmental duplications in the *Sinocyclocheilus* genomes were more than those in other fishes. However, we are not sure whether the results were due to chromosomal rearrangements or chromosome doubling.

Note S2

2. Genome annotation

2.1 Repetitive sequence detection

Almost every assembled genome is composed of repeated sequences. There are two main types of repeats in the genomes, including tandem repeats and interspersed repeats (such as LINEs and SINEs). We identified repetitive sequences or transposable elements (TEs) of three fishes based on combination of homology and *de novo* prediction methods using a variety of softwares [7].

We performed homology-based prediction by running the RepeatMasker (version 3.3.0) [8] with the default parameters against the Repbase [9], and using TE library (version 16.10) as well as RepeatProteinMask also with the default parameters at the DNA and protein levels to identify repeat sequences. For *de-novo* prediction of TEs, we searched the denovo-prediction-build repeat library using RepeatModeler (version 1.0.5) and generated TE results with classification information for each repeat family by running RepeatMasker on the genome sequences subsequently. Tandem repeats were also searched using the Tandem Repeats Finder (version 4.04) [10] with parameters "Match=2, Mismatch=7, Delta=7, PM=80, PI=10, Minscore=50, and MaxPeriod=2000". We generated the final results (Tables S10 to S12) with integrating all of the repeat annotation results using an in-house perl program. The sequence divergence rate was also calculated for each family of TEs (Figure S8).

2.2 Protein-coding gene annotation

The protein coding genes were obtained using combination of *de novo* method, homology-based gene prediction and RNA-seq data. All predicted gene evidence was integrated by GLEAN [11] to get non-redundant data [12].

For homology-based gene prediction, protein coding sequences of zebrafish, three-spined stickleback (*Gasterosteus aculeatus*), medaka, green spotted puffer (*Tetraodon nigroviridis*) and human (*Homo sapiens*) from Ensembl database (release 64) were used for analysis. The longest transcripts were chosen to represent the alternative splicing variants of genes [4]. Firstly, we aligned our assembled genomes to those protein sequences using TBLASTN (blast-2.2.23) [13] with E-value < 1e-5 and dealt with the alignment results by SOALR. Then GeneWise (version 2.2.0) [14] was used to predict the precise transcript structure and filter low-quality results with cds <150 bp after clustering the gene set. Therefore, the homology-based gene sets were obtained.

Before performing *de novo* prediction, we masked all the repetitive sequences in the assembled genomes. Then we carried out annotation using Augustus [15], SNAP [16] and GlimmerHMM

[17] with gene model parameters trained by 800 completed genes selected from homolog alignment, and finally obtained the intersection of results from the three methods.

In addition, RNA-seq data from different tissues including eye, skin, liver and gonad of three fishes were also used to verify the gene sets. First, we mapped transcriptome data to the genome using Tophat (v.2.0.4) [18], and then got transcriptome-based gene structures by cufflinks (v.2.0.0) [19]. Lastly, all gene structures obtained by the three approaches were combined using GLEAN to filter genes with low confidence level.

Using the above approaches, we obtained the final gene sets of the three *Sinocyclocheilus* species (Sg: 42,109 genes, Sr: 40,333 genes and Sa: 40,470 genes) (Table S13). CEGMA was used again to assess the coverage rate between KOG (EuKaryotic Orthologous Groups) genes predicted by CEGMA and the three gene sets in this paper (Table S14). The results indicate that the predicted gene sets cover more than 98% of KOGs at least. Meanwhile, gene numbers in each set are nearly as twice as the number of zebrafish (26,206), which supports that the *Sinocyclocheilus* lineages are tetraploid. The distributions in mRNA, CDS, exon and intron lengths of protein coding genes in each genome with a comparison to zebrafish are also summarized (Figure S9). Additionally, we identified co-linearity blocks between zebrafish and Sg genomes regarding adjacent synteny genes as a block. With more than half of blocks have 2:1 relationship between Sg and zebrafish (Figure S10), we infer that there was another genome duplication in *Sinocyclocheilus* (maybe at an unknown ancient node) which is called the 4R duplication event.

In order to further understand the species' biological adaptation, we explored their gene functions. First, we used our coding proteins blast against databases including PRINTS [20], PROSITE [21], Pfam [22], ProDom [23], SMART [24] and PANTHER [25] by InterProScan [26] to annotate motifs and domains. Meanwhile, GO (Gene Ontology) [27] items for each gene were gained from the InterProScan result. Then we also searched KEGG (Kyoto Encyclopedia of Genes and Genomes) databases [28] (release 58) to find the pathways in which the gene might be involved, as well as Swissprot (version 2012.03) and TrEMBL (version 2012.03) to confirm the gene symbols. The final results are listed in Table S15.

2.3 Non-coding RNA annotation

A non-coding RNA (ncRNA) is any RNA molecule that is not translated into a protein. Four types of ncRNA, including micro RNA (miRNA), transfer RNA (tRNA), ribosomal RNA (rRNA), and small nuclear RNA (snRNA) were annotated in our analysis. There are four types of rRNA composed of 5S, 5.8S, 18S and 28S in eukaryotes, which are quite conservative. Hence, we

searched rRNAs by blasting human rRNA database. As for siRNAs and snRNAs, we blasted against Rfam [29, 30] database which divided ncRNAs into families based on evolution from a common ancestor. Finally, we used tRNAscan-SE [31] to search reliable tRNAs in the genomes. The detailed information is shown in Tables S16 to S17.

Note S3

3. Transcriptome analysis

3.1 RNA isolation, and RNA-Seq method

RNA was isolated for sequencing from four tissues (eye, skin, liver, and ovary) of Sg, Sr and Sa respectively. RNA-seq libraries were constructed using the Illumina mRNA-Seq Prep Kit, then the libraries were sequenced using Illumina sequencing platform and the 90-bp paired-end sequencing module. About 4-Gb clean data were generated in each library.

3.2 RNA-Seq data processing

There are some adaptor sequences and/or low-quality reads presented in the raw reads. In order to obtain high-quality reads, we applied an in-house C++ program to filter our raw reads. The procedure included the following steps:

Remove reads with adaptor sequences.

Remove reads that contained > 10% ambiguous base calls (Ns).

Remove low-quality reads that contained > 40% low-quality base calls (quality score ≤ 5).

Remove read pairs with read 1 and read 2 overlapping by ≥ 10 bp and 100% identical read pairs.

3.3 Alignment and *de novo* assembly of the transcriptomes

All of the clean RNA-Seq reads were mapped onto the corresponding reference genomes (Sg, Sr and Sa) using TopHat. According to the mapped results, transcripts were constructed using cufflinks. The *de novo* transcriptomes of the four tissues were assembled by Trinity with filtered reads from each tissue separately into contigs and scaffolds. Trinity contains Inchworm, Chrysalis and Butterfly, which were employed sequentially to process large volumes of RNA-seq reads.

Note S4

4. Evolutionary analysis of *Sinocyclocheilus* genomes

4.1 Gene family cluster

Orthologs refer to homologous sequences derived by a speciation event from a single ancestral sequence in the last common ancestor of the examined species. We defined gene families using TreeFam (http://www.treefam.org) among the three *Sinocyclocheilus* species (Sg, Sr and Sa) and seven other vertebrates, including fugu, green-spotted puffer, three-spined stickleback, Atlantic cod, medaka, zebrafish, and human. First, we ran all-vs-all blast with the e-value of 1e-7 using ten species's protein sequences to rapidly find an initial list of possible matches. Then, we conjoined the blast alignments by Solar, removed genes with aligned ratio < 0.33, converted the bit score to percent score, and grouped the genes by hcluster_sg (https://pypi.python.org/pypi/hcluster, version 0.5.0). Eventually, 17,883 gene families and 210 single-copy gene families were generated. The numbers of orthologous genes across the ten species were counted (Figure S13, Table S19) and plotted in a Venn diagram (Figure S14).

4.2 Phylogenetic analysis

We constructed phylogenetic trees using the gene sets of Sg, Sr and Sa, together with seven other published vertebrate genomes (fugu, green-spotted pufferfish, three-spined stickleback, Atlantic cod, medaka, zebrafish and human). Since *Sinocyclocheilus* are tetraploid, the single-copy families were rather fewer than in other vertebrates. We extracted these genes with two-copy families in *Sinocyclocheilus* but one-copy in the other seven vertebrates, and then removed the shorter one of the two copies in *Sinocyclocheilus*, hence the remaining longer genes were presented as a group of single-copy families. Together with the original 210 single-copy families, we obtained 3,181 single-copy families. Subsequently, we extracted amino acid sequences from the 3,181 single-copy genes to concatenate one super-gene, and constructed phylogenetic trees using maximum likelihood (ML) method in PhyML [32, 33] and Bayes inference method in MrBayes [34]. Different datasets from whole coding sequences, 1st, 2nd, 1st & 2nd, 3rd and 4d codon positions were used to reconstruct phylogenetic tree respectively. All results revealed that Sg and Sa clustered together and then grouped with Sr with high bootstrap values.

Meanwhile, six mitochondrial gene (*COX1*, *COX2*, *COX3*, *ND3*, *ND4* and *ND6*) sequences were used to reconstruct the phylogenetic trees with the same pipeline and parameters as described above [35], which also supported the same phylogenetic relationships. These topologies of the species tree are shown in Figure S15 and S16.

4.3 Divergence time estimation

Based on the phylogenetic tree and sequences derived from CDS, 1st and 2nd and 4d codon positions, and using five fossil records (Takifugu-Tetraodon, 32.25~56 Ma; Stickleback-Takifugu, Tetraodon, 96.9~150.9 Ma; Medaka-Stickleback, Takifugu, Tetraodon, 96.9~150.9 Ma; Zebrafish-Medaka, Stickleback, Takifugu, Tetraodon, 149.85~165.2 Ma; Fishes-toad, bird, mammal, 416~421.75 Ma) [36] as calibrating time points, we estimated the species divergence time using the mcmctree [37] in PAML [38] with default values in parameter settings. Furthermore, we also recalculated the divergence time using the multidivtime [39, 40] program.

The generated chronogram using mcmctree in PAML based on 4d codon positions revealed that Sr emerged first from other two species at 26.3 Ma, and Sg divided from Sa at 17.5 Ma. We also obtained similar results when using other methods (Multidivtime, CDS sequences) and datasets (1st& 2nd) to estimate divergence times, which are shown in Figure S17.

4.4 Demographic history

The distribution of time to TMRCA (the most recent common ancestor) between two alleles in an individual can be related to the history of population size fluctuation. The population size histories of Sg, Sr and Sa were inferred using the pairwise sequentially Markovian coalescent (PSMC) model [41] on heterozygous sites with the generation time (g=1 year) (according to artificial breeding of Sg) and the mutation rate ($m=3.51 \times 10^{-9}$ per year per nucleotide) [42]. Reconstructed population history was plotted for Sg, Sr and Sa separately using gnuplot4.4 [43].

4.5 Gene family contraction and expansion

According to the above-mentioned phylogenetic trees and divergence time, we explored gene families that underwent a significant change in *Sinocyclocheilus* lineages. After filtering the families that have only one copy member or those have more than 200 copies in one species while other species had less than 2 copies, we performed *Sinocyclocheilus* lineage-specific expansion and contraction analysis using the CAFÉ program [44]. Based on a random birth and death model [45], a global parameter λ was estimated using maximum likelihood. Then, gene family numbers in each species and their ancestor were estimated. Comparing each branch and their ancestor branch, a conditional p-value was calculated and families with p-value less than 0.05 were marked as a significant change [46], which means it underwent contraction or expansion in the process of evolution (Figure 2a).

After that, the families that significantly change in size were subjected to GO/KEGG/IPR

enrichment analyses along each *Sinocyclocheilus* lineage, respectively (Table S24).

Among those gene families undergoing expansion and contraction, a contractive family *OPN5* in Sa (GO: 0007601: visual perception) caught our attention, because it may be associated with eye degeneration for cave adaption. Opsins are members of the guanine nucleotide-binding protein-coupled receptor superfamily. *OPN5*, which is expressed in the eye, brain, testes, and spinal cord, belongs to the seven-exon subfamily of mammalian opsin genes that includes peropsin (RRH) and retinal G protein coupled receptor (RGR) and encodes a protein with photoisomerase activity [47]. The olfactory receptor family is the largest one in the genome, responsible for the recognition and G protein-mediated transduction of odorant signals. We found the *OR* family (GO:0004984 olfactory receptor activity) has undergone a large expansion, especially *OR10*, *OR13*, *OR2*, *OR51*, *OR52*, *OR1* and *OR6*. There were some other families associated with immune response, such as *DPB1* family (GO:0042613 MHC class II protein complex) which have experienced contraction, while *HLA-B* family (GO:0042612 MHC class I protein complex) has experienced expansion.

More details are shown in Table S29 in additional file 2 (Table S29-genefam.expan.contract.list.xlsx)

4.6 Genes with accelerated evolutionary rates

Positive Darwinian selection at the DNA sequence level has been tested by estimating the ratio (ω) of nonsynonymous nucleotide substitutions (dN) to synonymous nucleotide substitutions (dS) between ortholog genes [48]. We performed positive selection analysis based on the single-copy genes that we used in the phylogenetic analysis. After filtering families with an alignment ratio of less than 0.4, we set the three *Sinocyclocheilus* species as the foreground branch respectively, while other species in our phylogenetic tree were set as the background branch. A branch-site model [49] was used to calculate different ω in phylogenetic tree, such as ω_0, ω_2 and ω_1 representing the whole tree, foreground branch and background branch respectively [35]. Then a p-value test was used to check whether ω_2 was significantly greater than ω_1 and ω_0. The genes with a p-value less than 0.05 implied that they may have undergone positive selection in the *Sinocyclocheilus* lineages.

After obtaining the *Sinocyclocheilus* positive selection gene (PSG) list, we converted it to the corresponding human orthologs as the input against a background of human genes [48] using DAVID Functional Annotation [50] tool. The annotation and enrichment result files are listed in Table S30 in additional file 3 (Table S30-positive.selection.list.xlsx).

In the Sa branch, the following genes should be given special attention: *DHX30*, *Rab3gap1*,

BBS4, *Gpr143* and *galE*. *DHX30* encodes a mitochondrial nucleotide protein which is identified as a component of a transcriptional repressor complex that functions in retinal development. Defects in *Rab3gap1* are the cause of Warburg micro syndrome 1 [51, 52], which is a severe autosomal recessive disorder characterized by developmental abnormalities of the eye and central nervous system. Defects in *BBS4* are the cause of Bardet-Biedl syndrome type 4 (BBS4) that is usually characterized by severe pigmentary retinopathy, early onset obesity, polydactyly, hypogenitalism, renal malformation and mental retardation [53]. *Gpr143* encodes a protein that binds to heterotrimeric G proteins and is targeted to melanosomes in pigment cells. Mutations in this gene cause ocular albinism type 1 [54], especially associated with visual impairment. Mutations in *galE* result in epimerase-deficiency galactosemia, also referred to as galactosemia type 3, a disease characterized by liver damage, early-onset cataracts, deafness and mental retardation [55]. All the above genes may be connected to physiological and morphological changes in cavefishes.

In the Sr branch, we also found *DHX30* and *Rab3gap1* underwent positive selection. Accordingly, we found *DGKI*, a member of type IV diacylglycerol kinase subfamily, may play a crucial role in the production of phosphatidic acid in the retina or in recessive forms of retinal degeneration (from NCBI, no reference). Mutations in *HPS1* may play a role in organelle biogenesis associated with melanosomes, platelet dense granules and lysosomes, and are associated with Hermansky-Pudlak syndrome type 1 which characterized by oculocutaneous albinism, bleeding and lysosomal storage defects [56]. *GIPC3* has also undergone positive selection; its mutations are associated with autosomal recessive deafness [57].

4.7 Evolution of Hox clusters

With the highly conserved sequences and synteny genomic architecture, the Hox clusters are usually used as evidence of whole genome duplication [58]. More importantly, the order of Hox genes along the chromosome reflects the order in which they act along the body. Teleost fishes are the pinnacle of Hox cluster evolution, with at least seven Hox clusters and 49 genes in zebrafish [58].

To define Hox genes in the three *Sinocyclocheilus* genomes, the Hox genes of zebrafish were downloaded from ensembl, then reciprocal best ortholog analysis was used with an e-value cutoff of 1e-5 and filtered with less than 80% of alignment and 75% overall amino acid identity.

The gene numbers for the *Sinocyclocheilus* species were nearly twice of those in zebrafish (Table S18). Remarkably, the order of Hox genes along the scaffolds in *Sinocyclocheilus* reflects the same order as in zebrafish, and the diagrams compared syntenic relationships with the Hox of

zebrafish linkage groups. The multiple copies of Hox genes indicated that *Sinocyclocheilus* fishes are indeed tetraploids.

Hox genes define patterns of development in vertebrate limbs. For each Hox cluster composed of Anterior (group 1,2), Group3 (group 3), Central (group 4~8) and Posterior (9~13) classes of Hox genes existed before the divergence of the protostome and deuterstome lineages. From the results (Figure S11), we found that the conserved non-coding sequences encompass a surprisingly large part of the clusters, especially in the *HoxBa* and *HoxCa* clusters.

Group 1 appeared in *HoxA*, *HoxB* and *HoxC* clusters of zebrafish and *Sinocyclocheilus* genomes, therefore it was used to construct a similarity network. The results (Figures S12A to S12B) showed that each of these three clusters of *Sinocyclocheilus* was orthologous to one of the zebrafish clusters. Hence, the duplication events of *Sinocyclocheilus* that produced the clusters occurred at least before the divergence of *Sinocyclocheilus* lineages at about 26.3 million years ago (Figure 2a).

4.8 Loss of Sa-specific gene families

In order to identify Sa-specific gene family loss, we extracted gene families that have no member in Sa while being larger than zero in the other nine species. The lost gene family list is included in Table S23. Many genes, such as *LMCD1*, *TEM-7*, *PDCD5*, *BCL6*, *F7*, *CA14*, *CYP2U1* and *Creb3l4*, have lost at least one copy in Sa. Previous studies have shown that *Creb3l4* can regulate the expression of genes required for germ cell survival but is insufficient to disrupt the normal fertility in mice [59]. We infer that the loss of *Creb314* may have some relationship with its low fecundity. The inner mitochondrial membrane protein Mpv17 in podocytes is essential for the maintenance of mitochondrial homeostasis and protects podocytes against oxidative stress-induced injury both in vitro and in vivo [60]. *LMCD1*, *TEM-7* (tumor endothelial marker 7), *PDCD5* were associated with tumor metastasis [61], antiaging [62] and enhanced apoptosis [63] respectively. *BCL6* may function in a 'hit-and-run' role in lymphomagenesis [64]. F7 (EC 3.4.21.21, blood-coagulation factor VIIa, activated blood coagulation factor VII, formerly known as proconvertin) is one of the proteins that causes blood to clot in the coagulation cascade. CA14 is a Carbonic anhydrase (CA) that catalyzes the reversible hydration of carbon dioxide. It participates in a variety of biological processes, including respiration, calcification, acid-base balance, bone resorption, and the formation of aqueous humor, cerebrospinal fluid, saliva, and gastric acid. It has shown extensive diversity in tissue distribution and in their subcellular localization [65]. *CYP2U1* (cytochrome P450, family 2, subfamily U, polypeptide 1) metabolized arachidonic acid, docosahexaenoic acid (DHA), and

other long chain fatty acids, suggesting a potential role in brain and immune functions [66].

TSC2 in Sa is under positive selection. Its gene product is believed to be a tumor suppressor and is able to stimulate specific GTPases, which is consistent with the gene loss of *LMCD1*, *TEM-7*, *PDCD5* for tumor suppression.

Note S5

5. Morphological comparison analysis

5.1 Paraffin section of eyes of the three *Sinocyclocheilus* species

Histological studies on eyes were performed according to the following procedures: (1) Fixation: Separated eye regions were fixed in 4% paraformaldehyde at 4℃ overnight. (2) Dehydration: Eye tissue was dehydrated through a series of alcohols, including 30%, 50%, 70%, 85%, two 95%, and three 100% steps, 20 min per step. They were transferred into ethanol/TO-regent (1:1) twice, and then moved into TO reagent twice more. (3) Embedment: Paraffin/TO-reagent (1:1) was added for one hour (62℃), followed by 100% paraffin twice in two hours (62℃). The tissue was embedded into melting and solidifying paraffin. (4) Sectioning: Tissues were sectioned at 5–15 μm thickness in the microtome blocks, and then spread the sections into glass slides. (5) Dewaxing the Slides and Rehydration of Tissues: Dewaxed slides were placed into two TO containers for ten minutes each, and then the rehydrated slides were placed into graded ethanol solutions: 100%, 95%, 90%, 80%, and 70% for 3-5 minutes each. (6) Staining: Sections were stained using Hematoxyli-Eosin (HE). (7) Photography: The sections were checked on the slides and photographed under a microscope.

5.2 Immunocytochemistry of taste buds

Immunocytochemistry analysis of taste buds (checking only the separated upper and lower jaws, hereafter called tissues) was carried out according to the following steps: (1) Fixation: Tissues were fixed in 4% paraformaldehyde in PBS overnight at 4℃. (2) Immunocytochemistry: The tissues were washed thoroughly in PBS and then permeabilized overnight at 4℃ in PBS containing 4% Triton X-100. (3) The tissues were washed thrice more the following day for 15 min each with PBS at room temperature. They were incubated in primary antibody (Rabbit against calretinin, labeling entire receptor cells within taste buds) and occasionally agitated gently in a dark chamber for 5-6 days at 4℃. This antibody was added together in PBS with 0.5% Triton X-100 to yield final dilutions of 1:1500. (4) The tissues were rinsed three times for 30 min in each with PBS. (5) The tissues were incubated in secondary antibodies (a final dilution of 1:500). Incubation in secondary antibody similarly occurred with gentle agitation in the dark chamber for further 2-3 days at 4℃. (6) The tissues were rinsed three times for 45 min each with PBS. (7) Negative controls were conducted in tissues that were incubated in secondary antibody without

first being labelled with primary antibody. (8) The tissues were viewed using a microscope equipped for epifluorescence.

5.3 Anatomy of gas bladder, absolute fecundity and other measurements

Samples for anatomy and measurements were from specimens immersed in 90% ethanol. We used dissecting scissors and tweezers to separate the gas bladder from other viscera and then took photos and measured them. Absolute fecundity was counted from the number of eggs in individuals at maturity stages IV (mature) or V (ripe). The counting of lateral line scales was conducted under a stereo microscope.

5.4 Synchrotron X-ray microtomography of the saccular otolith

The synchrotron X-ray microtomography experiments were performed at BL13W1 beamline of Shanghai Synchrotron Radiation Facility. A specimen was held in a vertical tube mounted on a sample stage. If the specimen was larger than the X-ray beam, the sample stage was moved vertically for continuous scans. Specimens were imaged with 13 keV monochromatic X-ray at 9 um resolution for larger specimens, and at 3.7 um for juvenile specimens. The slices were reconstructed using the FBP algorithm, and 3D renderings were created and manipulated in VGStudio 2.1 software.

Note S6

6. Cave adaption analysis

6.1 Vision analysis

Protein sequences of 140 zebrafish vision-related genes were downloaded from the present Ensembl database and aligned to our gene sets using blastp with E-value <1e-5. The homolog genes of the three *Sinocyclocheilus* species were determined according to the identity with zebrafish's category and the copy numbers were counted (Tables S25A to S25E). Multiple protein sequence alignments of homolog genes were performed with MUSCLE (vision 3.8.31) [67]. The results demonstrated that Sg *rcv-1* gene was premature and Sa *rom1b* gene has a two–amino-acid insertion and the inserted site is located in the Tetraspanin family conserved domain (also testified by PCR) [NCBI accession: pfam00335] (Figure S23). The sequencing depth near the insert site was found with IGV (Integrative Genomics Viewer) software (vision 2.3.32) (Figure S23).

A similarity cluster of crystalline genes of zebrafish and three *Sinocyclocheilus* species is shown in Figure S28. The *Sinocyclocheilus* genes were classified according to the zebrafish gene nomenclature. Among 60 crystallin genes, 29 zebrafish genes were defined as outgroup compared with *Sinocyclocheilus* genes, however, 4 zebrafish genes cannot be distinguished from those of the *Sinocyclocheilus* species. *Crygm2d* gene in the *Sinocyclocheilus* fishes cannot be classified into any particular category, although it was the closest to *Crygm2d12* of zebrafish (Figure S28).

Development and maintenance of photoreceptors requires a series of transcriptional factors including *Crx* (cone-rod homebox), *Nrl* (neural retina leucine zipper protein), *Otx2* (orthodenticle homolog-2), *Otx5* (orthodenticle homolog-5), *Nr2e3* (nuclear receptor subfamily 2 group E member 3), *Thrb* (thyroid hormone receptor beta), *Guca1a* (guanylate cyclase activator 1A), *Gnat1*, *Gnat2*, *Irbp* (interphotoreceptor retinoid-binding protein), *Rorb* (RAR related orphan receptor beta) [68]. Our eye transcriptome analysis of the three *Sinocyclochseilus* species showed that the expression levels of transcriptional factors were Sg>Sr>Sa (Tables S25A to S25E, S26A to S26D, Figures S25A to 25B). Ten transcriptional factors (*Crx*, *Nrl*, *Otx2*, *Otx5*, *Nr2e3*, *Ggca1A*, *Gnat1*, *Gnat2*, *Rorb*, *Rx3*) were significantly down-regulated in Sa compared with Sg and Sr. On the contrary, some intriguing non-vision direct related genes showed different expression patterns in the three species. For example, the expression (RPKM value) of *Irf6* (interferon regulatory factor 6) in Sa was 158.6, which was significantly higher than Sg (2.2) and Sr (0). *Irf6* is related to the formation of connective tissue and play a critical role in keratinocyte development [69]. Tubulins are a small family of globular proteins. The most common members of the tubulin

family are α-tubulin and β-tubulin which make up microtubules. The expression of tubulin alpha chain in Sa was 18.9 while there was no expression in Sg and Sr. These genes may be involved in formation of connective tissue and degeneration of eyes in the cave-restricted Sa.

Meanwhile, the expression of *peripherin2* (photoreceptor outer segment membrane glycoprotein 2) was detected in Sg (90.1) and Sr (1.5) while there was no expression in Sa. Secreted extracellular proteins are often glycosylated to be functional and we speculated that the function of photoreceptor outer segment membrane may decline in Sa. Oat (Ornithine aminotransferase) is an enzyme involved in the ultimate formation of proline from ornithine. A deficiency of this enzyme causes Oat deficiency, also known as gyrate atrophy of choroid and retina. The presenting symptom of Oat deficiency is myopia which progresses to night blindness [70]. No expression of *Oat* in Sa and Sr is consistent with the partial or entire dark environment. *Pde6h-b* is a gene that encode retinal rod rhodopsin-sensitive cGMP 3',5'-cyclic phosphodiesterase subunit gamma which is related to photosensitivity in rod photoreceptor cells [71]. The expression of *Pde6h-b* in Sa (6.3) was significantly lower than those in Sg (4174) and Sr (626). These observations may suggest different developmental genetic mechanisms of retinal degeneration occurred in the cavefish Sa.

6.2 Pigmentation

To define orthology genes related to melanogenesis in the three *Sinocyclocheilus* genomes, a blastp analysis was employed with the e-value cutoff of 1e-5 using Sg, Sr, Sa coding proteins, respectively. When setting human pigment protein genes as the reference, we picked the best hit for each pigment gene in the three species, and then filtered those with less than 50% of matching rate and less than 50% overall amino acid identity (along the entire sequence). Reciprocal best-match pairs were defined as orthologs. Multiple sequence alignments (MSA) were performed using MUSCLE.

Tyr (Tyrosinase) is a key rate-limiting enzyme in the melanin production. Mutations in this gene cause human oculocutaneous albinism 1. By comparing *Tyr* ortholog sequences, we found a nucleotide mutation (G419R) in one copy of Sa (also testified by PCR), which is identical with one of human mutations in *Tyr* (http://www.ifpcs.org/albinism/oca1mut.html). This mutation site has been first reported in Caucasian patients [72], then tested in Indo-Pakistani [73], Indian [74] and Non-Hispanic Caucasians [75] patients. The alignment results are presented in Figure S18.

We also checked melanin-associated-gene expression in the skin transcriptome of the three *Sinocyclocheilus* species. Compared to Sg, *Tyr* (P-value of 1.69E-05, FDR of 3.38E-05) and *Tyrp1* (P-value of 3.87E-67, FDR of 1.55E-66) in Sa was significantly down-regulated, while no

expression was detectable in Sr. Meanwhile, *Dct* expressed in Sr was significantly up-regulated (Table S20). The expression levels of genes in the melanogenesis pathways in Sa are the lowest.

6.3 Scale development

Edar (Ectodysplasin-A receptor) is a member of the Tumor necrosis factor receptor superfamily [76] which works together with other proteins during embryonic development and plays a key role in initiation of hair development in mammals. It encoded a protein with approximately 450 amino acids, including front signal peptide, extracellular region, transmembrane region and cytoplasmic region. Recently, Edar has been shown to be required for scale development in fishes [77].

We downloaded *Edar* encoded protein (*ENSDARP00000045289*) from the zebrafish genome and predicted two copies of *Edar* genes in the three *Sinocyclocheilus* genomes. Subsequently, we aligned these proteins using MUSCLE against *ENSDARP00000045289* and found deletions (Figure S20) in the *Edar* gene of Sa.

In our work, we found two copies of *Edar* genes in the genomes of the three *Sinocyclocheilus* species while both of the two copies in Sa present deletions (also testified by PCR) including signal peptide. These signal peptides guide new protein transfer across the membrane and parts of the extracellular region. The deletions may make the functional protein incomplete resulting in little scale covering on the skin surface of Sa (Figure S29).

6.4 Hearing

Using the same method as described in the pigment-related genes, we obtained a series of genes associated with deafness and ear development. MSA was executed to find unique amino-acid changes. *Mpv17* encodes a mitochondrial inner membrane protein associated with mitochondrial DNA depletion syndrome. It has been reported that a *Mpv17*-deficient mice can cause severe morphological degeneration of the cochlea, resulting in sensorineural hearing loss at 2-months-old [78]. We took out the best hit for *Mpv17* and found that one copy of this gene in Sa has a deletion in the signal region which may lead to the functional decline (Figure S22). *Ush2a* is another gene that altered significantly in Sa. Two amino-acid changes, R334S [79, 80] and V382A [81] (also shown by PCR) were predicted (Figure S19). It has been proved that the encoded protein is found in the basement membrane, and may be important in the development of the inner ear and retina. In addition, mutations within this gene have been associated with Usher syndrome type IIa and retinitis pigmentosa (https://grenada.lumc.nl/LOVD2/Usher_montpellier/home.php?select_db=USH2A).

A frequent polymorphism in the translation start codon of Otoraplin (*Otor*) can abolish translation and may be associated with forms of deafness. The encoded protein has growth-inhibitory activity in melanoma cell lines [82]. Since we detected *Otor* in Sr, Sa and *Astyanax mexicanus*，the *Otor* may have an impact on hearing and pigment development in the cavefishes. Interestingly, the reconstructions of the saccular otolith morphology using synchrotron X-ray microtomography (Figure 4), as well as both the anatomy of the swim bladder (Figure S30) and distributions of the neuromasts of the trunk lateral line system (Figure S31), indicated that these hearing related organs in Sa presented different degrees of weakness compared to Sg and Sr. Furthermore, a comparison to the distribution of neuromasts on the head (Figure 4) also hinted that the response to vibration of these three species was Sg>Sr>Sa.

6.5 Immune response

Protein sequences of 2,157 human immune response genes were downloaded from GO database. A blsatp analysis was used with the e-value cutoff of 1e-5 to identify homolog genes in zebrafish and the three *Sinocyclocheilus* species. Then, the following steps were used to filter those candidate genes: a) deleted genes with identity and align rate less than 30%; b) executed GO enrichment and removed genes that are not immune-related GO terms; c) discarded genes that appeared with many immune-related GO terms. At last, we acquired the specific information of immune genes of each *Sinocyclocheilus* species (Table S21).

The immune ability of cavefishes was suspected to be lower than surface fishes. The *Tlr* gene family is a group of innate immune specific receptors. We analyzed the *Tlr* gene family among the three *Sinocyclocheilus* species and zebrafish, and found that *Tlr* 5 in Sa is contracted (Table S22). We performed similarity analysis with eleven sequenced fishes and representative species of four branches (mammals: human and mouse, birds: chicken, amphibians: toad, reptile: anolis and turtle) (Figure S27). The data showed that the fishes' *Tlr* copy numbers are greater than those found in the other branches, and the *Sinocyclocheilus Tlr* genes are closer to those of zebrafish, which is consistent with their close phylogenomic relationship.

6.6 Circadian rhythm

Previous research has shown that cavefishes lack circadian rhythms because they live in perpetual darkness [83]. Mexican blind cavefish, *Astyanax mexicanus*, has a phenotype with reduced sleep in comparison to surface populations [84]. We checked the rhythm pathway gene

copy number and their expressions in the semi-cave fishes Sr and cavefish Sa. Related zebrafish genes were downloaded from the KEGG pathway database (map 04710) and aligned to the gene sets of the three *Sinocyclocheilus* species. The homolog genes of these species were determined according to the identity with zebrafish's category and their copy numbers were counted (Table S27). Homolog protein sequence multiple alignments were performed with MUSCLE and the structural variations were validated. The data demonstrated that both copies of Skp1 protein in Sa were deleted in the N-terminal (Figure S24) (also testified by PCR). It was reported that Skp1 protein serves as an adapter in SCF complex, which links the F-box protein to Cul1 and plays an important role in the maintenance of animal circadian rhythm. Meanwhile, transcriptomic analysis showed that the rhythm genes' expression levels in the three *Sinocyclocheilus* species declined in the order Sg>Sr>Sa (Figure S26). These observations together suggest a weak circadian rhythm in the cavefish Sa.

6.7 Sense of taste

There are five primary tastes: sweet, bitter, salty, sour and umami. Peptides of Tas1r1 and Tas1r3 form a heterodimer to detect the umami taste, whereas peptides of Tas1r2 and Tas1r3 form a heterodimer for sweet detection. *Tas2r* genes are responsible for the bitter taste [85]. Furthermore, *ENaC* and *Pkd2l1*, channels responsible for sensing salty and sour tastes, respectively. In the three *Sinocyclocheilus* genomes, the copy numbers of *Tas1r3* and *Pkd2l1* were double those in the zebrafish genome. In particular, the copy number of *Tas1r2* (responsible for sweet) was more than double compared with zebrafish, suggesting that the *Sinocyclocheilus* fishes were more sensitive to sweet (Table S28). The *ENaC* gene was not identified in the three *Sinocyclocheilus* genomes and this may suggest that *Sinocyclocheilus* fishes have lost the ability to taste salt.

In conclusion, the genomic data suggest that the *Sinocyclocheilus* fishes are likely able to sense umami, sweet, bitter, and sour tastes, but not salty, which is consistent with their current freshwater environment.

References (1-85)

1. Zhao Y, Zhang C. Endemic Fishes of Sinocyclocheilus (Cypriniformes: Cyprinidae) in China-Species diversity, Cave adaptation, Systematics and Zoogeography. Beijing: Science Press; 2009.
2. Li R, Fan W, Tian G, Zhu H, He L, Cai J et al. The sequence and de novo assembly of the giant panda genome. Nature. 2010; 463(7279):311-7.
3. Li R, Yu C, Li Y, Lam T-W, Yiu S-M, Kristiansen K et al. SOAP2: An improved ultrafast tool for short read alignment. Bioinformatics. 2009; 25(15):1966-7.
4. Wang Z, Pascual-Anaya J, Zadissa A, Li W, Niimura Y, Huang Z et al. The draft genomes of soft-shell turtle and green sea turtle yield insights into the development and evolution of the turtle-specific body plan. Nat Genet. 2013; 45(6):701-6.
5. Parra G, Bradnam K, Korf I. CEGMA: a pipeline to accurately annotate core genes in eukaryotic genomes. Bioinformatics. 2007; 23(9):1061-7.
6. Parra G, Bradnam K, Ning Z, Keane T, Korf I. Assessing the gene space in draft genomes. Nucleic Acids Res. 2009; 37(1):289-97.
7. Jiang Y, Xie M, Chen W, Talbot R, Maddox JF, Faraut T et al. The sheep genome illuminates biology of the rumen and lipid metabolism. Science. 2014; 344(6188):1168-73.
8. Tarailo-Graovac M, Chen N. Using RepeatMasker to identify repetitive elements in genomic sequences. Curr Protoc Bioinformatics. 2009; Chapter 4:Unit 4.10.
9. Jurka J, Kapitonov VV, Pavlicek A, Klonowski P, Kohany O, Walichiewicz J. Repbase Update, a database of eukaryotic repetitive elements. Cytogenet Genome Res. 2005; 110(1-4):462-7.
10. Benson G. Tandem repeats finder: a program to analyze DNA sequences. Nucleic Acids Res. 1999; 27(2):573-80.
11. Elsik CG, Mackey AJ, Reese JT, Milshina NV, Roos DS, Weinstock GM. Creating a honey bee consensus gene set. Genome Biol. 2007; 8(1):R13.
12. Cho YS, Hu L, Hou H, Lee H, Xu J, Kwon S et al. The tiger genome and comparative analysis with lion and snow leopard genomes. Nat Commun. 2013; 4:2433.
13. Altschul SF, Gish W, Miller W, Myers EW, Lipman DJ. Basic local alignment search tool. J Mol Biol. 1990; 215(3):403-10.
14. Birney E, Clamp M, Durbin R. GeneWise and Genomewise. Genome Res. 2004; 14(5):988-95.
15. Stanke M, Keller O, Gunduz I, Hayes A, Waack S, Morgenstern B. AUGUSTUS: ab initio prediction of alternative transcripts. Nucleic Acids Res. 2006; 34(Web Server issue):W435-9.
16. Korf I. Gene finding in novel genomes. BMC Bioinformatics. 2004; 5:59.
17. Majoros WH, Pertea M, Salzberg SL. TigrScan and GlimmerHMM: two open source ab initio eukaryotic gene-finders. Bioinformatics. 2004; 20(16):2878-9.
18. Trapnell C, Pachter L, Salzberg SL. TopHat: discovering splice junctions with RNA-Seq. Bioinformatics. 2009; 25(9):1105-11.
19. Trapnell C, Hendrickson DG, Sauvageau M, Goff L, Rinn JL, Pachter L. Differential analysis of gene

regulation at transcript resolution with RNA-seq. Nat Biotechnol. 2013; 31(1):46-53.
20. Attwood TK, Beck ME. PRINTS--a protein motif fingerprint database. Protein Eng. 1994; 7(7):841-8.
21. Hulo N, Bairoch A, Bulliard V, Cerutti L, De Castro E, Langendijk-Genevaux PS et al. The PROSITE database. Nucleic Acids Res. 2006; 34(Database issue):D227-30.
22. Finn RD, Mistry J, Tate J, Coggill P, Heger A, Pollington JE et al. The Pfam protein families database. Nucleic Acids Res. 2010; 38(Database issue):D211-22.
23. Bru C, Courcelle E, Carrere S, Beausse Y, Dalmar S, Kahn D. The ProDom database of protein domain families: more emphasis on 3D. Nucleic Acids Res. 2005; 33(Database issue):D212-5.
24. Letunic I, Doerks T, Bork P. SMART 7: recent updates to the protein domain annotation resource. Nucleic Acids Res. 2012; 40(Database issue):D302-5.
25. Mi H, Lazareva-Ulitsky B, Loo R, Kejariwal A, Vandergriff J, Rabkin S et al. The PANTHER database of protein families, subfamilies, functions and pathways. Nucleic Acids Res. 2005; 33(Database issue):D284-8.
26. Zdobnov EM, Apweiler R. InterProScan--an integration platform for the signature-recognition methods in InterPro. Bioinformatics. 2001; 17(9):847-8.
27. Harris MA, Clark J, Ireland A, Lomax J, Ashburner M, Foulger R et al. The Gene Ontology (GO) database and informatics resource. Nucleic Acids Res. 2004; 32(Database issue):D258-61.
28. Kanehisa M, Goto S, Shuichi K, Akihiro N. The KEGG databases at GenomeNet. Nucleic Acids Res. 2002; 30(1):42-6.
29. Griffiths-Jones S, Bateman A, Marshall M, Khanna A, Eddy SR. Rfam: an RNA family database. Nucleic Acids Res. 2003; 31(1):439-41.
30. Gardner PP, Daub J, Tate JG, Nawrocki EP, Kolbe DL, Lindgreen S et al. Rfam: updates to the RNA families database. Nucleic Acids Res. 2009; 37(Database issue):D136-40.
31. Lowe TM, Eddy SR. tRNAscan-SE: a program for improved detection of transfer RNA genes in genomic sequence. Nucleic Acids Res. 1997; 25(5):955-64.
32. Guindon S, Dufayard JF, Lefort V, Anisimova M, Hordijk W, Gascuel O. New algorithms and methods to estimate maximum-likelihood phylogenies: assessing the performance of PhyML 3.0. Syst Biol. 2010; 59(3):307-21.
33. Guindon S, Delsuc F, Dufayard JF, Gascuel O. Estimating maximum likelihood phylogenies with PhyML. Methods Mol Biol. 2009; 537:113-37.
34. Huelsenbeck JP, Ronquist F. MRBAYES: Bayesian inference of phylogenetic trees. Bioinformatics. 2001; 17(8):754-5.
35. Zhang G, Cowled C, Shi Z, Huang Z, Bishop-Lilly KA, Fang X et al. Comparative analysis of bat genomes provides insight into the evolution of flight and immunity. Science. 2013; 339(6118):456-60.
36. Donoghue PC, Benton MJ. Rocks and clocks: calibrating the Tree of Life using fossils and molecules. Trends Ecol Evol. 2007; 22(8):424-31.
37. Yang Z. PAML 4: phylogenetic analysis by maximum likelihood. Mol Biol Evol. 2007; 24(8):1586-91.

38. Yang Z. PAML: a program package for phylogenetic analysis by maximum likelihood. Comput Appl Biosci. 1997; 13(5):555-6.
39. Wikstrom N, Savolainen V, Chase MW. Evolution of the angiosperms: calibrating the family tree. Proc Biol Sci. 2001; 268(1482):2211-20.
40. Wang ZQ. A new Permian gnetalean cone as fossil evidence for supporting current molecular phylogeny. Ann Bot. 2004; 94(2):281-8.
41. Li H, Durbin R. Inference of human population history from individual whole-genome sequences. Nature. 2011; 475(7357):493-6.
42. Graur D, Li W-H. Fundamentals of molecular evolution. Sinauer Associates Sunderland; 2000.
43. Philipp JK. Gnuplot in Action: Understanding Data with Graphs. Manning Publications; 2009.
44. De Bie T, Cristianini N, Demuth JP, Hahn MW. CAFE: a computational tool for the study of gene family evolution. Bioinformatics. 2006; 22(10):1269-71.
45. Hahn MW, De Bie T, Stajich JE, Nguyen C, Cristianini N. Estimating the tempo and mode of gene family evolution from comparative genomic data. Genome Res. 2005; 15(8):1153-60.
46. Demuth JP, De Bie T, Stajich JE, Cristianini N, Hahn MW. The evolution of mammalian gene families. PLoS One. 2006; 1:e85.
47. Yamashita T, Ono K, Ohuchi H, Yumoto A, Gotoh H, Tomonari S et al. Evolution of mammalian Opn5 as a specialized UV-absorbing pigment by a single amino acid mutation. J Biol Chem. 2014; 289(7):3991-4000.
48. Sun YB, Zhou WP, Liu HQ, Irwin DM, Shen YY, Zhang YP. Genome-wide scans for candidate genes involved in the aquatic adaptation of dolphins. Genome Biol Evol. 2013; 5(1):130-9.
49. Zhang J, Nielsen R, Yang Z. Evaluation of an improved branch-site likelihood method for detecting positive selection at the molecular level. Mol Biol Evol. 2005; 22(12):2472-9.
50. Huang da W, Sherman BT, Lempicki RA. Systematic and integrative analysis of large gene lists using DAVID bioinformatics resources. Nat Protoc. 2009; 4(1):44-57.
51. Abdel-Salam GM, Hassan NA, Kayed HF, Aligianis IA. Phenotypic variability in Micro syndrome: report of new cases. Genet Couns. 2007; 18(4):423-35.
52. Dursun F, Guven A, Morris-Rosendahl D. Warburg Micro syndrome. J Pediatr Endocrinol Metab. 2012; 25(3-4):379-82.
53. Riise R, Tornqvist K, Wright AF, Mykytyn K, Sheffield VC. The phenotype in Norwegian patients with Bardet-Biedl syndrome with mutations in the BBS4 gene. Arch Ophthalmol. 2002; 120(10):1364-7.
54. Ohtsubo M, Sato M, Hikoya A, Hosono K, Minoshima S, Hotta Y. Case of Japanese patient with x-linked ocular albinism associated with GPR143 gene mutation. Jpn J Ophthalmol. 2010; 54(6):624-6.
55. Bang YL, Nguyen TT, Trinh TT, Kim YJ, Song J, Song YH. Functional analysis of mutations in UDP-galactose-4-epimerase (GALE) associated with galactosemia in Korean patients using mammalian GALE-null cells. FEBS J. 2009; 276(7):1952-61.

56. Sandrock K, Bartsch I, Rombach N, Schmidt K, Nakamura L, Hainmann I et al. Compound heterozygous mutations in 2 siblings with Hermansky-Pudlak syndrome type 1 (HPS1). Klin Padiatr. 2010; 222(3):168-74.

57. Charizopoulou N, Lelli A, Schraders M, Ray K, Hildebrand MS, Ramesh A et al. Gipc3 mutations associated with audiogenic seizures and sensorineural hearing loss in mouse and human. Nat Commun. 2011; 2:201.

58. Meyer A, Van de Peer Y. From 2R to 3R: evidence for a fish-specific genome duplication (FSGD). Bioessays. 2005; 27(9):937-45.

59. Adham IM, Eck TJ, Mierau K, Muller N, Sallam MA, Paprotta I et al. Reduction of spermatogenesis but not fertility in Creb3l4-deficient mice. Mol Cell Biol. 2005; 25(17):7657-64.

60. Casalena G, Krick S, Daehn I, Yu L, Ju W, Shi S et al. Mpv17 in mitochondria protects podocytes against mitochondrial dysfunction and apoptosis in vivo and in vitro. Am J Physiol Renal Physiol. 2014; 306(11):F1372-80.

61. Chang CY, Lin SC, Su WH, Ho CM, Jou YS. Somatic LMCD1 mutations promoted cell migration and tumor metastasis in hepatocellular carcinoma. Oncogene. 2012; 31(21):2640-52.

62. Bagley RG, Rouleau C, Weber W, Mehraein K, Smale R, Curiel M et al. Tumor endothelial marker 7 (TEM-7): a novel target for antiangiogenic therapy. Microvasc Res. 2011; 82(3):253-62.

63. Murshed F, Farhana L, Dawson MI, Fontana JA. NF-kappaB p65 recruited SHP regulates PDCD5-mediated apoptosis in cancer cells. Apoptosis. 2014; 19(3):506-17.

64. Green MR, Vicente-Duenas C, Romero-Camarero I, Long Liu C, Dai B, Gonzalez-Herrero I et al. Transient expression of Bcl6 is sufficient for oncogenic function and induction of mature B-cell lymphoma. Nat Commun. 2014; 5:3904.

65. Kuijpers MJ, van der Meijden PE, Feijge MA, Mattheij NJ, May F, Govers-Riemslag J et al. Factor XII Regulates the Pathological Process of Thrombus Formation on Ruptured Plaques. Arterioscler Thromb Vasc Biol. 2014; 34(8):1674-80.

66. Chuang SS, Helvig C, Taimi M, Ramshaw HA, Collop AH, Amad M et al. CYP2U1, a novel human thymus- and brain-specific cytochrome P450, catalyzes omega- and (omega-1)-hydroxylation of fatty acids. J Biol Chem. 2004; 279(8):6305-14.

67. Edgar RC. MUSCLE: multiple sequence alignment with high accuracy and high throughput. Nucleic Acids Res. 2004; 32(5):1792-7.

68. Meng F, Braasch I, Phillips JB, Lin X, Titus T, Zhang C et al. Evolution of the eye transcriptome under constant darkness in Sinocyclocheilus cavefish. Mol Biol Evol. 2013; 30(7):1527-43.

69. Blanton SH, Cortez A, Stal S, Mulliken JB, Finnell RH, Hecht JT. Variation in IRF6 contributes to nonsyndromic cleft lip and palate. Am J Med Genet A. 2005; 137A(3):259-62.

70. Bangal S, Bhandari A, Dhaytadak P, Gogri P. Gyrate atrophy of choroid and retina with myopia, cataract and systemic proximal myopathy: A rare case report from rural India. Australas Med J. 2012; 5(12):639-42.

71. Pittler SJ, Baehr W, Wasmuth JJ, McConnell DG, Champagne MS, vanTuinen P et al. Molecular characterization of human and bovine rod photoreceptor cGMP phosphodiesterase alpha-subunit and chromosomal localization of the human gene. Genomics. 1990; 6(2):272-83.
72. King RA, Mentink MM, Oetting WS. Non-random distribution of missense mutations within the human tyrosinase gene in type I (tyrosinase-related) oculocutaneous albinism. Mol Biol Med. 1991; 8(1):19-29.
73. Tripathi RK, Bundey S, Musarella MA, Droetto S, Strunk KM, Holmes SA et al. Mutations of the tyrosinase gene in Indo-Pakistani patients with type I (tyrosinase-deficient) oculocutaneous albinism (OCA). Am J Hum Genet. 1993; 53(6):1173-9.
74. Chaki M, Sengupta M, Mukhopadhyay A, Subba Rao I, Majumder PP, Das M et al. OCA1 in different ethnic groups of india is primarily due to founder mutations in the tyrosinase gene. Ann Hum Genet. 2006; 70(Pt 5):623-30.
75. Hutton SM, Spritz RA. Comprehensive analysis of oculocutaneous albinism among non-Hispanic caucasians shows that OCA1 is the most prevalent OCA type. J Invest Dermatol. 2008; 128(10):2442-50.
76. Monreal AW, Ferguson BM, Headon DJ, Street SL, Overbeek PA, Zonana J. Mutations in the human homologue of mouse dl cause autosomal recessive and dominant hypohidrotic ectodermal dysplasia. Nat Genet. 1999; 22(4):366-9.
77. Kondo S, Kuwahara Y, Kondo M, Naruse K, Mitani H, Wakamatsu Y et al. The medaka rs-3 locus required for scale development encodes ectodysplasin-A receptor. Curr Biol. 2001; 11(15):1202-6.
78. Muller M, Smolders JW, Meyer zum Gottesberge AM, Reuter A, Zwacka RM, Weiher H et al. Loss of auditory function in transgenic Mpv17-deficient mice. Hear Res. 1997; 114(1-2):259-63.
79. Dreyer B, Tranebjaerg L, Rosenberg T, Weston MD, Kimberling WJ, Nilssen O. Identification of novel USH2A mutations: implications for the structure of USH2A protein. Eur J Hum Genet. 2000; 8(7):500-6.
80. Baux D, Larrieu L, Blanchet C, Hamel C, Ben Salah S, Vielle A et al. Molecular and in silico analyses of the full-length isoform of usherin identify new pathogenic alleles in Usher type II patients. Hum Mutat. 2007; 28(8):781-9.
81. Garcia-Garcia G, Aparisi MJ, Jaijo T, Rodrigo R, Leon AM, Avila-Fernandez A et al. Mutational screening of the USH2A gene in Spanish USH patients reveals 23 novel pathogenic mutations. Orphanet J Rare Dis. 2011; 6:65.
82. Robertson NG, Heller S, Lin JS, Resendes BL, Weremowicz S, Denis CS et al. A novel conserved cochlear gene, OTOR: identification, expression analysis, and chromosomal mapping. Genomics. 2000; 66(3):242-8.
83. Cavallari N, Frigato E, Vallone D, Frohlich N, Lopez-Olmeda JF, Foa A et al. A blind circadian clock in cavefish reveals that opsins mediate peripheral clock photoreception. PLoS Biol. 2011; 9(9):e1001142.
84. Duboue ER, Keene AC, Borowsky RL. Evolutionary convergence on sleep loss in cavefish populations.

Curr Biol. 2011; 21(8):671-6.
85. Yarmolinsky DA, Zuker CS, Ryba NJ. Common sense about taste: from mammals to insects. Cell. 2009; 139(2):234-44.
